# Transient Absorption Spectroscopy of Blue Copper Sites in Divergent Protein Folds: Azurin Versus Multicopper Oxidase

**DOI:** 10.1002/cphc.202500924

**Published:** 2026-04-28

**Authors:** Luis Ignacio Domenianni, Patrycja Kielb

**Affiliations:** ^1^ Clausius Institute of Physical and Theoretical Chemistry University of Bonn Wegelerstrasse 12 Bonn Germany; ^2^ Transdisciplinary Research Area ‘Building Blocks of Matter and Fundamental Interactions’ University of Bonn Bonn Germany

**Keywords:** blue copper protein, impulsive stimulated Raman scattering, protein fold effects, ultrafast dynamics, vibrational coherence

## Abstract

Blue copper proteins share a highly conserved type‐1 copper coordination that gives rise to characteristic spectroscopic signatures, yet they are embedded in evolutionarily divergent protein folds. Here, we investigate how differences in protein architecture modulate ultrafast electronic and vibrational relaxation following optical excitation of the blue copper site. Using transient absorption spectroscopy, we compare the excited‐state dynamics of two evolutionarily distant proteins: the single‐domain electron‐transfer protein azurin and two‐domain small laccase (SLAC) belonging to the family of multicopper oxidases. Kinetic analysis of the transient spectra reveals coherent vibrational wave packets generated via impulsive stimulated Raman scattering, providing access to low‐frequency collective modes associated with the electronic ground state. Notably, distinct dominant modes are observed for the two proteins, centered at 38 cm^−1^ for SLAC and 29 cm^−1^ for azurin. These differences are correlated with variations in structural rigidity and coordination constraints beyond the first coordination spheres of the blue copper site. Our results reveal that, despite conserved metal coordination, the surrounding protein arrangement plays a significant role in shaping ultrafast energy relaxation pathways in metalloprotein.

## Graphical abstract

Ultrafast pump‐probe spectroscopy of two evolutionarily divergent blue copper proteins reveals distinct low‐frequency ground‐state collective modes (38 cm^−1^ in SLAC, 29 cm^−1^ in azurin) generated via impulsive stimulated Raman scattering, potentially reflecting the differences in the rigidity of the surrounding protein scaffold beyond a conserved Cu coordination sphere.

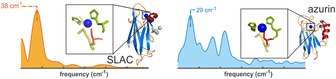

## 1 Introduction

Blue copper sites are ubiquitous metalloenzyme cofactors found across all biological kingdoms [1, 2, 3]: plants (plastocyanin), bacteria (azurin, laccases), fungi (laccases), and mammals (ceruloplasmin). They own their name to the unique and conserved coordination of the Cu ion by cysteine's sulfur, formally named copper type 1 (T1), and its overall tetrahedral geometry, which results in a distinctive spectroscopic signature and blue color [4]. Although their first‐sphere coordination is evolutionarily preserved, they are embedded within diverse protein folds [5]. They all emerge from the ancient cupredoxin‐like fold [6], an eight‐stranded Greek key β‐barrel [7], but diverged into different families of proteins of primarily two different functions: electron transfer proteins, built from a single cupredoxin domain, and oxidases built from multiple cupredoxin‐like domains, which evolved to couple electron transfer to oxygen reduction [5]. The evolutionary pressure for diverse function of the metal cofactor led to an adaptation toward new protein folds resulting in low overall sequence similarity among the blue copper proteins [8].

The steady‐state spectroscopies and electrochemistry have established broad similarities among coordination of blue copper sites, that is, distorted tetrahedral geometry of Cu‐coordinated by cysteine (Cys), two histidine (His) residues, and in most cases methionine (Met), absorbance peak at ca. 600 nm [4], three prominent peaks in the resonance Raman (RR) spectrum having strong Cu–S(Cys) stretching character [9], a small hyperfine coupling of ca. A_II_ ~220–230 MHz and g_II_ ~2.2–2.3 [10, 11, 12]. While all these features indicate a strong covalent bond of Cu–S(Cys), and result in redox potential in the range of 300–480 mV [13, 14], which are relevant in performing electron transfer, comparatively little is known about how the surrounding protein matrix modulates the functional properties of the copper cofactor*.* In turn, ultrafast spectroscopic techniques provide access to the excited‐state dynamics, revealing vibrational relaxation and protein motion beyond the first coordination sphere of the metal. Ultrafast pump‐probe spectroscopy has been employed to study blue copper proteins, revealing common dynamical motifs. Specifically, early femtosecond studies on plastocyanin [15, 16] demonstrated that excitation into the ligand‐to‐metal charge transfer (LMCT) band triggers an ultrafast decay of the excited state on a ~200–300 fs timescale, attributed to rapid nonradiative charge recombination from the copper center back to the ligand framework. Subsequent broadband pump–probe investigations on systems such as rusticyanin [17], plastocyanin [15, 16, 18], and ceruloplasmin [15] further highlighted the role of coherent vibrational dynamics, with oscillatory signals assigned to both localized active‐site modes (above 200 cm^−1^) and low‐frequency collective motions at 30–38 cm^−1^ vaguely assigned to collective protein motions [17]. Although ultrafast spectroscopies have been vastly employed to study excited state dynamics of different blue copper proteins [15, 16, 17, 18, 19], the energy relaxation pattern across the evolutionary divergent proteins has not been examined. Furthermore, direct comparison of previously reported individual blue copper proteins is complicated by differences in excitation conditions and analysis protocols. A systematic investigation under identical experimental conditions is therefore required to isolate structural contributions to ultrafast dynamics.

**FIGURE 1:**
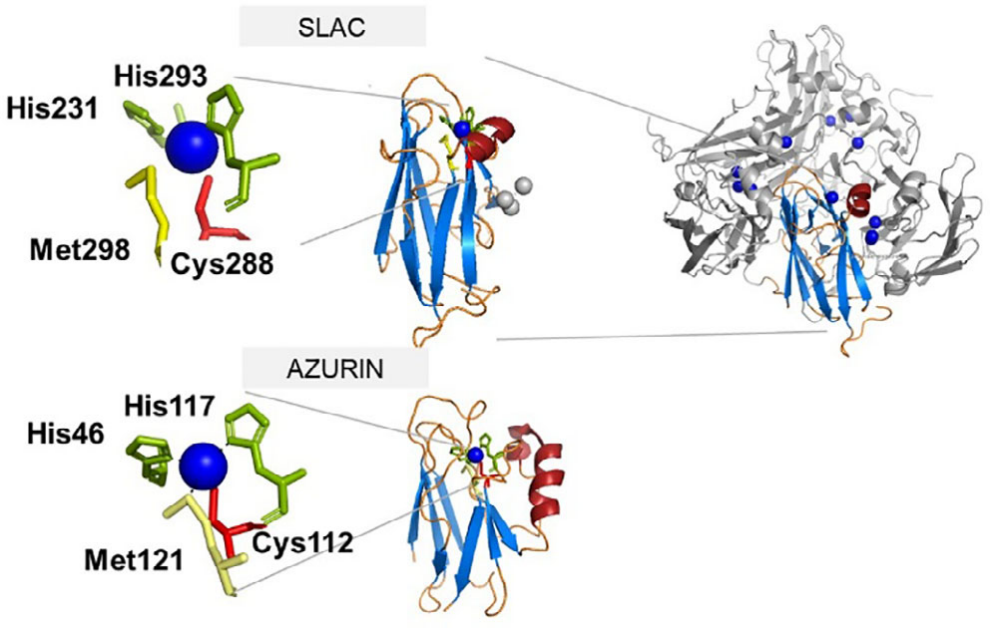
Blue copper site in azurin (top, pdb id: 1azu) and small laccase (bottom), coordinated by two His residues, Cys and Met residue. The protein fold colored by structural elements (β‐sheet – blue, α‐Helix – dark red, and random coil—orange) is shown to the right. SLAC (pdb id: 3cg8) is a homotrimer, in which each monomer consists of two domains: one housing blue copper site and the second domain which helps in coordinating remaining trinuclear copper site in between domains.

In this work, we address how the protein fold surrounding an otherwise conserved blue copper site modulates ultrafast electronic and vibrational relaxation following optical excitation. To this end, we compare the excited‐state dynamics of two evolutionarily distant blue copper proteins: the single‐domain electron‐transfer protein azurin from *Pseudomonas aeruginosa* [20] and the two‐domain multicopper oxidase laccase from *Streptomyces coelicolor* (SLAC) [12, 21, 22] (Figure 1). While azurin functions primarily as an electron shuttle between protein partners, small laccase (SLAC) couples substrate oxidation at the type‐1 copper site to the four‐electron reduction of oxygen to water at a distant trinuclear copper cluster. Structurally, azurin consists of a single cupredoxin domain, whereas SLAC comprises two cupredoxin‐derived domains [23].

Although both proteins share the characteristic cupredoxin fold—an eight‐stranded β‐barrel—their domain organization and secondary‐structure placement differ substantially (Figure 1 and S1). In particular, an α‐helix that directly approaches the copper site in SLAC provides a histidine ligand (His293), whereas in azurin all coordinating ligands originate from loop regions and a hydrophobic patch [24]. As a result, the two proteins represent distinct evolutionary solutions for embedding an otherwise conserved blue copper site within different protein matrices. We employ transient absorption spectroscopy (TAS) to probe the femtosecond‐to‐picosecond dynamics of metal‐centered and charge–transfer states and to elucidate how the surrounding protein matrix shapes nonequilibrium relaxation pathways. Analysis of the transient signals reveals coherent vibrational wavepacket motion generated via impulsive stimulated Raman scattering (ISRS) [25], enabling us to access low‐frequency collective modes that are not readily observable by conventional resonance Raman spectroscopy. While these modes cannot be unambiguously assigned to specific structural motions, their marked differences between azurin and SLAC demonstrate that ultrafast energy relaxation at blue copper sites is strongly influenced by the protein fold beyond the second coordination sphere.

## 2 Results and Discussion

### 2.1 UV/Vis Stationary Spectroscopy of SLAC and Azurin

**FIGURE 2:**
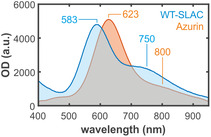
UV–Vis spectra of Azurin (orange) and SLAC (blue) dissolved in ammonium acetate and phosphate buffer, respectively.

The absorption spectra of both Azurin and SLAC recorded in the 400–950 nm range exhibit characteristic peaks for blue copper sites (Figure 2), that is, an intense, well‐resolved absorption band with a maximum at 583 nm for SLAC and 623 nm for Azurin, with a peak extinction coefficient on the order of 4500 – 4800 M^−1^ cm^−1^ [21, 26]. These strong absorption bands are responsible for their characteristic blue color and are attributed to a S‐Cys_pπ_‐>Cu^2+^d_x2‐y2_ ligand to metal charge transfer (LMCT) transition from the coordinating cysteine ligand to the Cu^2+^ ion [4, 9, 27]. UV/Vis spectra of both proteins exhibit a shoulder at low‐energy edge, with peak extinction coefficients of the order of 1000 M^−1^ cm^−1^ for Azurin and 2000 M^−1^ cm^−1^ for SLAC. These features, typical of copper complexes, are commonly assigned to partly forbidden transitions from filled d orbitals to the half‐occupied d_x2‐y2_ level [4]. The relatively high oscillator strength of the transition may arise from intensity borrowing from the LMCT band due to the shared symmetry of the d_xz,yz_ and the S–Cys(π) orbitals [28].

### 2.2 TAS of SLAC and Azurin

**FIGURE 3:**
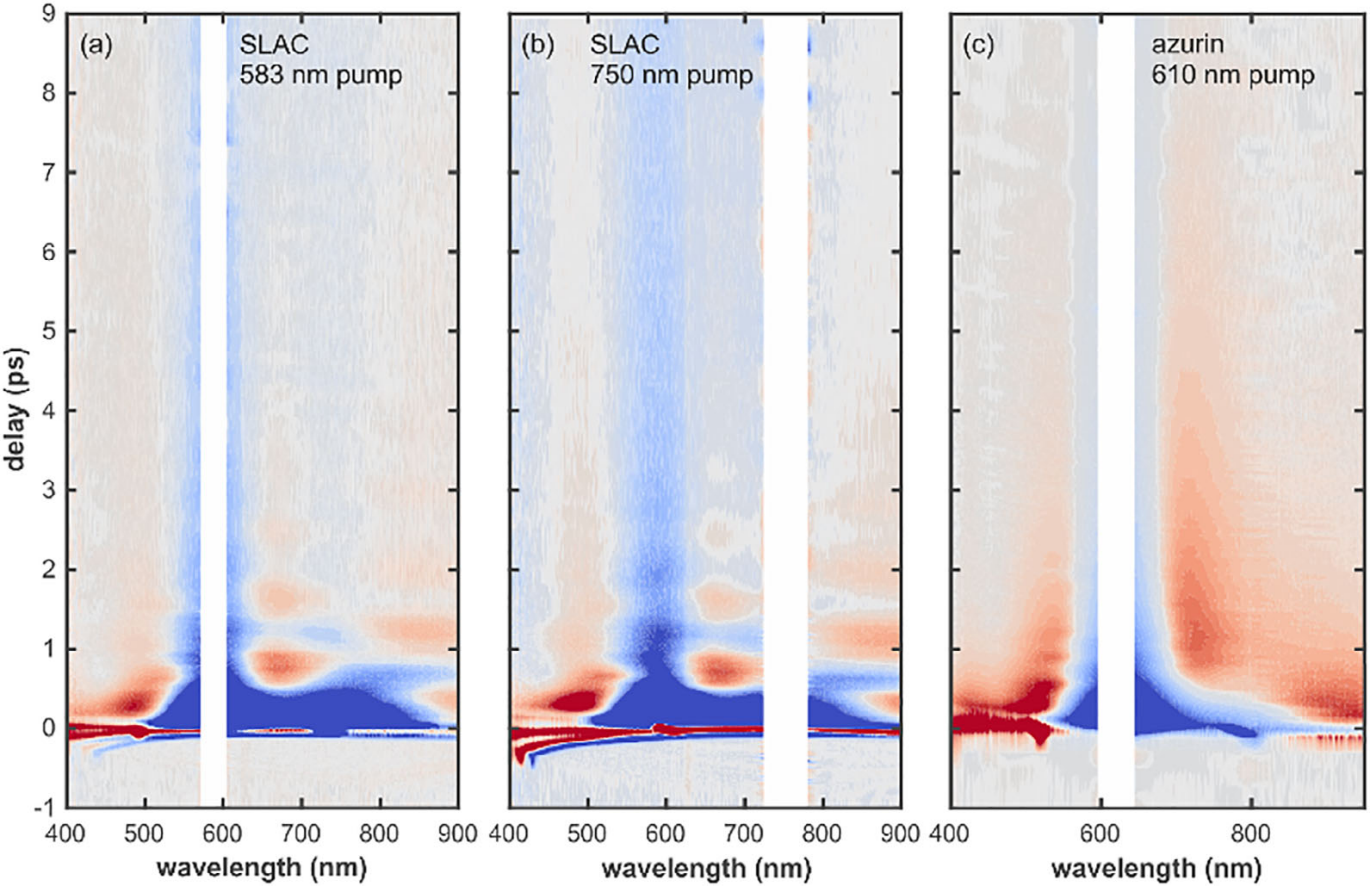
Contour plots representing transient visible spectra of (A) SLAC pumped with 583 nm, (B) SLAC pumped with 750 nm, and (C) Azurin pumped with 620 nm, as a function of time on ps timescale. Positive absorption bands are marked in red, while negative contributions due to bleach or stimulated emission are marked in blue.

**FIGURE 4:**
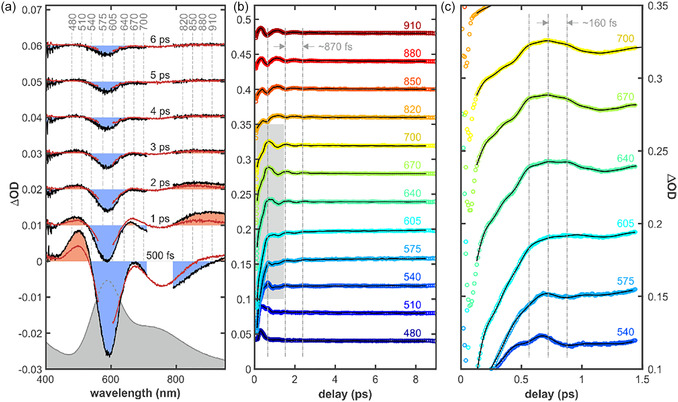
(a) Transient absorption spectra of SLAC at selected pump–probe delays. Black curves show experimental data recorded after 750 nm excitation, red curves represent the corresponding observed transient spectra after 583 nm excitation. For clarity, positive and negative spectral regions are shaded in light red and blue respectively, only for data recorded after 750 nm excitation. Vertical dashed lines mark selected probe wavelengths used for kinetic traces. (b and c) Transient absorption kinetic traces (ΔOD) measured at selected probe wavelengths following 750 nm photoexcitation. Open circles represent experimental data, while solid black lines indicate HSVD fits. Traces are vertically offset for clarity, and colors correspond to the indicated probe wavelengths (in nm). Vertical dash‐dotted lines were added to aid visualization of characteristic time periods. (b) Full time window up to ~ 9 ps, showing prompt population dynamics slowly modulated on the sub‐picosecond timescale (~870 fs). c) Early‐time dynamics (≤1.5 ps) highlighting the coherent wavepacket dynamics with a ~ 160 fs.

**FIGURE 5:**
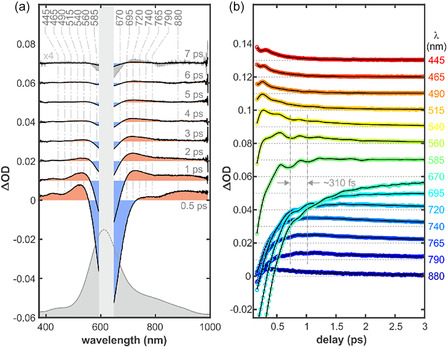
(a) Transient absorption spectra of azurin following 620 nm photoexcitation at selected pump‐probe delays and (b) corresponding transient absorption kinetic traces measured at selected probe wavelengths. Open circles represent experimental data, while solid black lines indicate HSVD fits. Traces are vertically offset for clarity, and colors correspond to the indicated probe wavelengths (in nm).

Ultrafast TAS spectroscopy is a powerful method to investigate fs‐to‐ps dynamics of metal‐centered and CT states, as shown on examples of transition metal compIlexes [29], or metalloproteins [30], including blue copper proteins [15, 17, 18]. By tracking the evolution of electronic populations and associated vibrational coherences, visible‐to‐NIR TA spectroscopy provides a detailed map of the relaxation pathways following impulsive excitation. In our work, we selectively excited the LMCT transitions of SLAC and azurin, centered at 583 and 620 nm, respectively, as well as d–d transition in SLAC, centered at ca. 750 nm. The obtained transient visible spectra are represented as contour plots of change in absorbance along the pump‐probe delay time, where positive induced absorption bands are depicted in red while negative contributions consisting of ground state bleach and/or stimulated emission from an excited state are depicted in blue (Figure 3). In all three cases, strong pump scattering is present and has been omitted from the spectra for clarity (Figure 3, blank space). After a short coherent artifact near time zero, both proteins exhibit pronounced oscillatory features modulating the transient signals. These features, indicative of nuclear wavepacket motion, appear as periodic variations in the transient intensity [25]. While the slowly evolving background components represent the population dynamics of the system, the complex oscillatory residuals provide a direct probe of vibrational coherences evolving on either the ground‐ or excited‐state potential energy surfaces (PES), which we analyze in detail in the following sections. Although the global evolution of the transient signals is summarized in Figure 3, the specific oscillatory components and their associated periods are more readily observed and discussed in the context of Figures 4 and 5 (vide infra).

The contour plot representations of the transient spectra of SLAC after 583 and 750 nm excitation are remarkably similar (Figure 3A,B), implying that the system follows the same trajectory through the same low‐lying states after optical excitation. The biggest difference is the reduced modulation depth at early delay times for optical excitation at 583 nm with respect to 750 nm. A similar behavior has been reported previously for plastocyanin by Nagasawa et al. [18]. following excitation at 590 and 890 nm, although time resolution differences between excitation wavelengths limit a quantitative comparison. Moreover, the visual inspection of SLAC's contour plots reveals at least two distinct oscillatory components: (i) a low‐frequency oscillation with a period of ca. 1 ps, accompanied by a clear phase flip around 750 nm and (ii) a high‐frequency oscillatory component with a time period of around 200 fs. The contour plots of the transient spectra of azurin (Figure 3C) represent different behavior. The transient spectra of azurin appear to be modulated only by a single frequency component of approximately 300 fs. The presence of an additional, strongly damped low‐frequency mode in azurin cannot be excluded, however, as similar behavior has been reported previously for related systems [18].

### 2.3 Kinetic Analysis of Transient Absorption Spectra—SLAC

Having qualitatively compared the contour plots of transient spectra between SLAC and azurin, we analyzed the time evolution of the optical features more closely. Immediately after photoexcitation of SLAC, the transient spectrum is characterized by *two pronounced* negative features centered at approx. 590 and 750 nm, coincidental with the peak intensities of the two absorption bands also observed in the linear UV–vis spectrum (Figure 4A). These features originate from the pump‐induced depletion of the electronic ground state and are therefore assigned to ground‐state bleach (GSB). In addition, a positive excited‐state absorption (ESA) band centered near 500 nm is also present at early times, arising from an electronically excited state of SLAC. The presence of yet another optically positive induced absorption (IA) band (within a range of 600–800 nm) can be inferred by the shape mismatch between the GSB and the inverted stationary UV–vis absorption spectrum of the sample (Figure 4A)*.*

On a time scale of ~1 ps (Figure 4A) the IA band centered at 500 nm decays almost completely, while two IA‐bands emerge: one centered at 870 nm and another one located in between the two GSB‐bands at 660 nm. After 6 ps, the negative bands recover to roughly 10% of their maximal amplitude (i.e., at a delay of 500 fs), and no other IA bands remain discernible. The overall spectral shape remains constant from 3 to 6 ps, with only the signal amplitudes evolving in time, indicating that the observed dynamics are governed by population relaxation rather than the formation of distinct transient species. Closer inspection of the kinetic traces around the IA bands and at the edges of the GSB reveals that at least two exponential components are required to adequately reproduce the data (Figure S5). While kinetic traces recorded near the main GSB bands (550 and 620 nm) display biexponential decay dynamics, the traces obtained at 660 and 870 nm exhibit an initial fast exponential rise followed by a slower exponential decay. The kinetic trace recorded at 500 nm shows a similar rise–decay behavior, although the rise seems to occur on a substantially faster timescale (Figure S5). Two scenarios could explain these observations. First, following optical excitation, an initially prepared excited state undergoes ultrafast relaxation and branches into two distinct excited‐state populations. The observed biexponential recovery of the GSB could therefore arise from parallel decay pathways of these two excited‐state populations. Secondly, the same observations could be explained by a simple sequential relaxation process, allowing for spectral overlap between the bleach and an intermediate species spectra. In fact, Nagasawa et al. [18] proposed that the deactivation kinetics of the optically prepared LMCT state in plastocyanin to a nearby metal center (MC) state occurs within the time range of ~40 fs, followed by ultrafast relaxation to lower LF states with a time constant of 90 fs. Later, relaxation to a hot ground state occurs with a time constant of ca. 250 fs, followed by vibrational cooling of the hot ground state with a time constant of ca. 1.8 ps, according to Equation (1).

(1) $$\mathrm{GS}\overset{h\nu }{\to }\text{LMCT}\overset{>40fs}{\to }\mathrm{MC}\overset{90fs}{\to }\mathrm{LF}\overset{250fs}{\to }{\mathrm{GS}}^{*}\overset{1.8ps}{\to }\mathrm{GS}$$

This model has the advantage of providing a simple explanation for the observation of identical dynamics following optical excitation at two different pump energies, since the d_*xy*_ → d_*x*_^2^−*y* ^2^ transition produces directly the MC state within time resolution and the follow up dynamics are identical.

To decompose contributions from the kinetic traces into population dynamics and coherent wavepacket motion, the transient signals were modeled as a sum of exponential decays and damped oscillatory components according to Equation (2)

(2) $$\Delta \mathrm{OD}\left(t\right)=\sum_{k=1}^{N}c_{k}\exp\left[\left(\frac{1}{\tau k}+i\omega_{k}\right)t+i\varphi_{k}\right]$$

from which amplitudes *c*_*k*_, decay/decoherence constants *τ*_k_, oscillation frequencies *ω*_k_, and phases φk are directly extracted using a Hankel singular value decomposition (HSVD) [31, 32].

Selected transient absorption kinetic traces measured at different probe wavelengths following 750 nm photoexcitation are shown in Figure 4B,C along with their respective HSVD fits. Frequency‐binned statistics with a bin width of 5 cm^−1^ revealed a maximum occurrence at 37.5 cm^−1^ for both excitation wavelengths (excluding the lowest frequency bin), in good agreement with the observed modulations with a time‐period of approx. 870 fs (38.3 cm^−1^) (Figure 4B). The mean decoherence time associated to the 37.5 cm^−1^ mode is 0.73 ps for both excitation wavelengths. In the early‐time (≤1.5 ps) dynamics (Figure 4C), an additional modulation of the transient absorption signal with a time period of approximately 160 fs is observed. Analysis of the zero‐frequency components of the HSVD fit reveals that the slowly varying background of the transient absorption signals can be accurately reproduced by four exponential components with time constants of about 350, 500 fs, 2  and 5.8 ps after both 583 and 750 nm pump.

To further confirm the kinetic model (Equation (1) ) we performed a global target analysis [33], using the HSVD time constants as initial guesses. To reduce the number of free parameters, we used the known stationary linear absorption spectrum to account for the GSB contribution following the procedure we have previously reported [34]. The target fit analysis yields rate constants, *k*_1_ = 1/[250 fs], k_2_ = 1/[350 fs], and k_3_ = 1/[4.3 ps] (k_3_ = 1/[5.1 ps] for dataset with 580 nm excitation). These constants describe the time‐dependent occupations of the four proposed states (Figure S9d) after 750 nm optical excitation of SLAC. The target fit analysis also yields the spectra associated with each state (Figure S9 (b) 583 nm pump and (c) 750 nm pump). The primary excited‐state, prepared within time resolution following optical excitation, exhibits two induced absorption bands both blue‐ and red‐shifted with respect to the ground‐state spectra. In contrast, both intermediate states resemble SLAC´s UV–vis spectra, though they appear broader and slightly blueshifted. This spectral signature is a characteristic hallmark of a vibrationally hot ground state, further supporting our assignment. Although vibrational cooling of small molecules in aqueous solution is expected to occur on the ~1 ps timescale, in proteins vibrational energy dissipation is mediated by the protein matrix and hydration shell, leading to multipicosecond cooling dynamics [35]. The observed 2 and 5 ps components are therefore consistent with thermalization of the ground state rather than distinct electronic intermediates.

### 2.4 Kinetic Analysis of Transient Absorption Spectra – Azurin

We have analyzed the time evolution of the transient spectra of azurin using the same approaches as described above for SLAC's spectra. At the earliest pump–probe delays (0.5 ps in Figure 5A), the transient spectrum is dominated by a pronounced negative pump‐induced optical density signal. Comparison of this feature with the linear vis–NIR absorption spectrum of azurin indicates that it originates from pump‐induced depletion of the electronic ground state. Strong pump scatter prevents a precise determination of the GSB minimum, which is estimated to lie between 590 and 640 nm. In addition, positive ESA bands are observed at early times on both the blue and red edges of the GSB, with absorption maxima near 510 and 880 nm, respectively, arising from electronically excited states. A minimum near 800 nm coincides with a secondary absorption feature in the linear spectrum. The spectral response in this region therefore, results from the overlap of transient absorption and GSB signals.

The IA bands centered at 510 and 880 nm decay almost completely within the first 3 ps (Figure 5). Concurrently, a new IA band emerges with a peak absorption centered at 710 nm. After 6 ps, the negative bands recover to approximately 1% of their maximum amplitude, while the remaining IA band decays concomitantly. This correlated behavior suggests that the observed dynamics are governed by population relaxation rather than by the formation of distinct transient species during this delay interval, as in SLAC. The slowly varying background of the transient absorption signals can be characterized by two exponential components with time constants centered around 350 fs and 1.8 ps, both exhibiting broad distributions, as indicated by statistical analysis of the zero‐frequency components from the HSVD fit. However, kinetic traces recorded near the main GSB band at 620 nm show biexponential recovery dynamics, while traces obtained at 540 and 740 nm display an initial fast exponential rise, followed by a slower exponential decay. These observations are in line with the results obtained for SLAC and could manifest returns of the initially photoexcited LMCT excited state to the ground state through several intermediate states, followed by subsequent vibrational cooling of the hot ground state [18].

Selected transient absorption kinetic traces measured at different probe wavelengths following 620 nm photoexcitation of azurin are shown in Figure 5B along with their respective HSVD fits. Frequency‐binned statistics with a bin width of 5 cm^−1^ revealed a maximum occurrence at 29 cm^−1^ corresponding to a time period of 1150 fs. Although an oscillation of such period has is not observed in the time traces, critically damped low‐frequency oscillations of similar values have been observed in the past by Nagasawa et al. for plastocyanin at 30 cm^−1^ [18]. A secondary maximum occurs at a frequency of 110 cm^−1^, in good agreement with the observed modulations with a time‐period of approx. 310 fs. The mean decoherence time associated to the 29 cm^−1^ mode is 550 fs.

The target fit analysis of azurin's transient spectra (after removing high‐frequency components identified via HSVD) with two intermediate steps reproduces the experimental data satisfactorily and yields rate constants, k1 = 1/[160 fs], k2 = 1/[235 fs] and k3=[1/1.97 ps], in good agreement with the values predicted by the HSVD. The time‐dependent normalized occupations of the proposed states as well as their spectra are shown in Figure S10. The assignment of the bands is done following the work of Nagasawa et al. [18]. The optically prepared state after excitation exhibits a small band centered at 450 nm and a long tail extending to the red with respect to the parent's linear spectra. The first intermediate state, presumably a MC d state, shows two bands peaking at 530 and 720 nm, while the last intermediate state resembles azurin´s vis‐NIR spectrum, albeit slightly broader, which supports the assignment to a vibrationally hot ground state.

### 2.5 Derived ISRS Spectra of SLAC and Azurin

**FIGURE 6:**
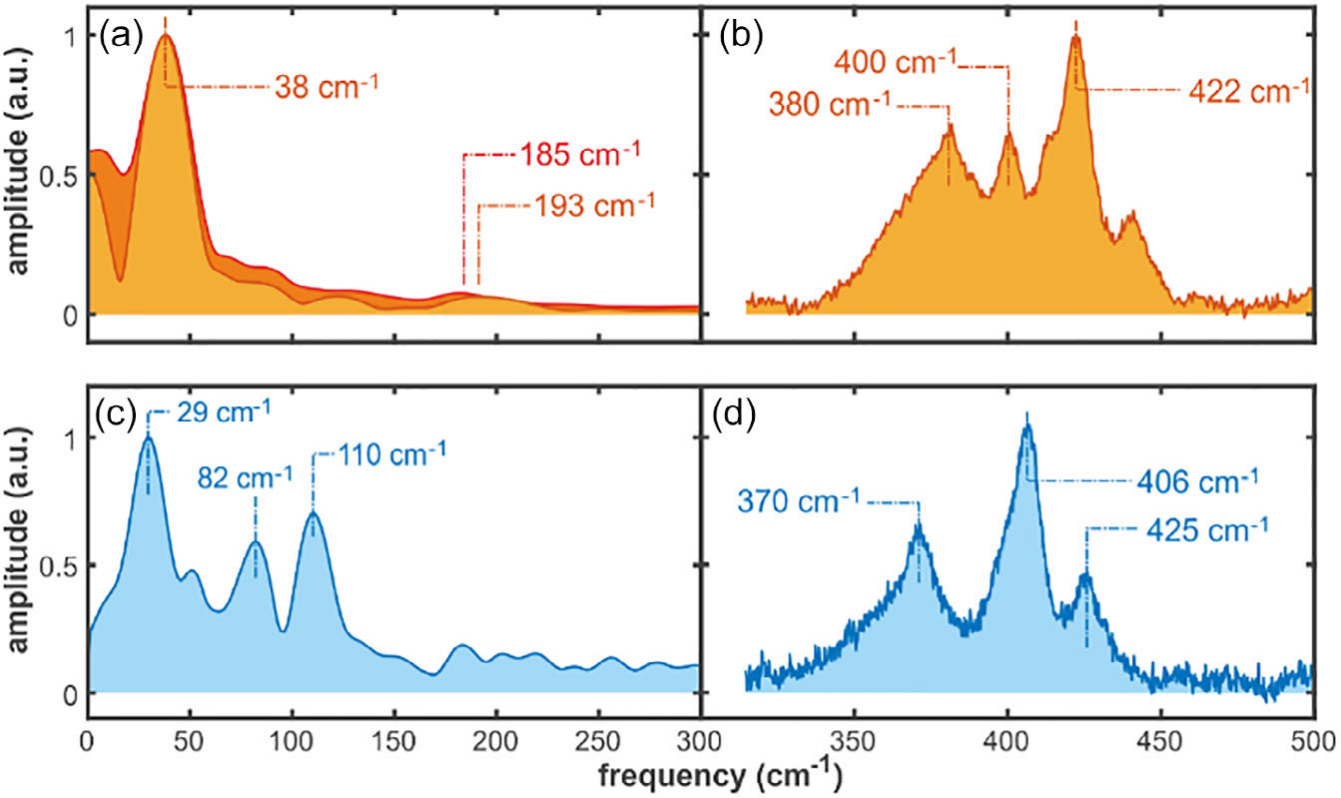
(a and c) Frequency‐domain ISRS spectra and (b and d) RR spectra of SLAC (top) and azurin (bottom). (a) Dark orange and light orange curves in a represent the amplitudes of the Fourier transformed spectra of the time‐dependent residual transient absorption signal of SLAC excited at 583 and 750 nm respectively. Both Fourier transformed spectra presented here are the average of the corresponding Fourier transformed spectra resulting from the probe wavelengths between 450 and 850 nm. The resulting ISRS spectra after excitation at 583 and 750 nm present similar features, dominated by a low‐frequency peak observed at 38 cm^−1^. Weaker higher‐frequency components extend into the 150–250 cm^−1^ range, being the highest observed bands –limited by the instrument time‐resolution‐ located at ~ 185 cm^−1^ (583 nm excitation) and ~ 193 cm^−1^ (750 nm excitation). (c) Frequency‐domain amplitudes by Fourier transformation of the time‐dependent residual transient absorption signal of azurin excited at 620 nm. The Fourier transformed spectra presented here is the average of the corresponding Fourier transformed spectra resulting from the probe wavelengths between 450 and 850 nm. RR spectra of (b) SLAC and (d) Azurin recorded using 594 nm laser line at 5 mW.

As the last step, the nonoscillatory (near zero‐frequency) components, associated with population decay and\or static offsets, were subsequently removed for each wavelength, and a Fourier transform was applied to the residual signal on the delay dimension. As such, the extracted vibrational frequencies and phases can be compared more reliably between pump frequencies, ensuring that any observed differences reflect genuine changes in wavepacket dynamics rather than artifacts of population decay or baseline offsets. The resulting frequency‐domain spectra of SLAC for both 583 and 750 nm excitation wavelengths are remarkably similar (Figure 6A). The spectra are dominated by a low‐frequency vibration observed at 38 cm^−1^ in good agreement with the HSVD results (38.3 cm^−1^), with a FWHM of 23 cm^−1^ which is slightly larger than the expected 15 cm^−1^ from the 0.73 ps time constant previously reported. Several weaker higher‐frequency components extend into the 150–250 cm^−1^ range, being the highest observed bands –limited by the instrument time‐resolution‐ located at ~185 cm^−1^ (583 nm excitation) and ~193 cm^−1^ (750 nm excitation). In contrast, in Azurin, the removal of the near zero‐frequency components and subsequent Fourier transformation of the residuals renders the frequency‐domain spectra (Figure 6C) dominated by a low‐frequency vibration observed at 29 cm^−1^ with a slightly larger than a shoulder band peaking at 55 cm−^1^, followed by two bands centered at 82 and 110 cm^−1^, in excellent agreement with the HSVD fit. For the comparison, the resonance Raman (RR) spectra of SLAC (Figure 6B) and azurin (Figure 6D) in the lowest detectable frequency region, excited with a 594 nm laser line, exhibit modes in the range of 250–422 cm^−1^. Three modes at 380 cm^−1^ in SLAC (370 cm^−1^ in Azurin), 400 (406 cm^−1^) and 422 cm^−1^ (425 cm^−1^) have been routinely assigned to the vibrations having partly Cu–S(Cys) stretch character, with the mode at ca. 400 cm^−1^ having its highest contribution [9, 36]. The slight deviations in frequency of this mode reflect the bond differences in the Cu—S bond length, with the higher frequency associated with the shorter bond, as also expected from the crystal structures of SLAC and azurin (Cu–S(Cys): 2.2 Ǻ ‐ SLAC, 1.8 Ǻ ‐ azurin).

As previously mentioned, the observed coherent oscillations may originate from vibrational wavepacket dynamics evolving either on the ground‐state or on the excited‐state PES. Formally, pump–probe spectroscopy is a third‐order nonlinear process, in which the system undergoes three light–matter interactions [37]. When the pump pulse duration is short relative to the vibrational period, called the impusive limit, the first two interactions prepare a coherent superposition of vibrational eigenstates, effectively launching a vibrational wavepacket that evolves on the excited‐state PES. Alternatively, the first pump interaction can create a coherent polarization on the excited‐state PES that evolves during a short period of time (smaller than the duration of the pump pulse) until the second pump interaction projects the system back to the ground state producing a ground‐state wavepacket displaced from equilibrium. This latter pathway produces coherent vibrational motion on the ground‐state PES through a resonant ISRS process [38]. To disentangle contributions from the excited and ground states, and to distinguish fundamental modes from potential cross‐mode beating, an analysis of the modulation frequency, amplitude and phase with respect to excitation wavelength is typically carried out [38, 39]. We have carefully inspected the oscillation amplitudes, phases, and frequency distributions associated with the dominant low‐frequency modes in both SLAC and azurin to ensure the assignments correspond to intrinsic vibrational coordinates.

**FIGURE 7:**
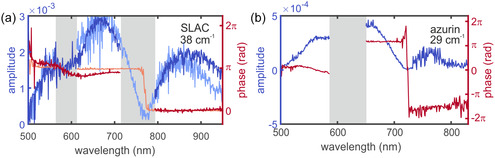
Spectrally resolved amplitude and phase retrieved from Fourier transformation of the residuals after removing slow varying background. Blue traces (left axis) show the signal amplitude, while red traces (right axis) show the corresponding phase (in radians). (a) Lowest detected frequency mode of SLAC, 38 cm^−1^. Lighter‐colored traces represent data obtained after optical excitation at 583 nm, while darker traces represent data obtained after optical excitation at 750 nm. (b) Lowest detected frequency mode of azurin, 29 cm^−1^.

In SLAC, we examine two low‐frequency modes: 38 cm^−1^ and 190 cm^−1^. For both modes, the oscillation amplitude drops to zero near the max of the ground‐state absorption band (at 750 nm), while the phase undergoes an abrupt shift of approximately π radians in the same spectral region (see Figure 7). Such observations are characteristic of a vibrational wavepacket evolving from the ground‐state PES [39], for which a minimal amplitude is expected at the absorption maximum and a π phase change between signals detected on the red and blue sides of the absorption band. Another remarkable observation is that the excitation wavelength does not seem to have an impact on this behavior. The assignment of the low frequency modes to the ground state is also consistent with the much longer decoherence time of such mode, 0.73 ps as obtained from the HSVD fit, than the observed lifetime of the excited state, 0.3 ps. As seen in the right panel of Figure 4B the 870 fs oscillations extend to ~3 ps, much longer than the excited state lifetime as can be seen clearly in Figure S9d.

In azurin, we examine three low‐frequency modes: 29, 82, and 110 cm^−1^ (Figure 7b and Figure S8, note that the shaded gray region centered around 620 nm marks the spectral range where strong pump scatter dominates, preventing reliable extraction of phase and amplitude information). Across all three frequency components, the oscillation amplitude exhibits two pronounced minima as a function of wavelength. Each minimum is accompanied by an abrupt, step‐like change in phase occurring at approximately the same spectral position, although the phase shift does not reach a full π jump. Such behavior is characteristic of dispersive responses arising from interference between multiple contributing pathways. The first amplitude minimum and associated phase discontinuity occur near the pump‐scatter region (~620 nm) and coincide with the maximum of the linear absorption spectrum. Although direct observation of the amplitude minima is hindered by pump scatter, the consistent phase offset observed on either side of this region, together with its spectral alignment with the absorption maximum, strongly suggests that the hinted phase discontinuity reflects a genuine physical effect rather than an artifact. A second amplitude minimum and phase change are observed near ~720 nm. This feature does not correspond to a maximum in the ground‐state absorption spectrum but lies close to a maximum associated with the MC excited state as identified in the target analysis. The differing spectral alignment of the two nodes indicates that they arise from distinct physical contributions. As such, the node near ~620 nm is most consistent with modulation dominated by ground‐state–linked pathways, such as GSB or impulsive Raman coherences, for which an amplitude minimum and phase inversion are expected at resonance. In contrast, the ~720 nm node, lacking correspondence with ground‐state absorption, is more naturally attributed to excited‐state–associated contributions, such as modulations of excited‐state absorption or stimulated emission. In this case, the observed phase behavior reflects interference between excited‐state pathways rather than a single dominant resonance. Notably, the phase change at 720 nm does not reach exactly π, indicating that no single pathway fully dominates the signal at this wavelength; instead, ground‐ and excited‐state contributions interfere, leading to a partial phase inversion. The wavelength dependence of the amplitude and phase thus reflects a superposition of coherences whose relative weights vary across the spectrum, rather than a purely ground‐ or excited‐state wavepacket. Consistent with this interpretation, the vibrational decoherence times extracted from HSVD analysis (550 fs) exceed the excited‐state population lifetime (~350 fs) obtained from target analysis. This reveals that the observed oscillations are not limited by excited‐state decay and supports the presence of long‐lived vibrational coherence, with a substantial contribution from ground‐state wavepacket motion.

### 2.6 Comparison Between Structural Folds of Azurin and SLAC

**FIGURE 8:**
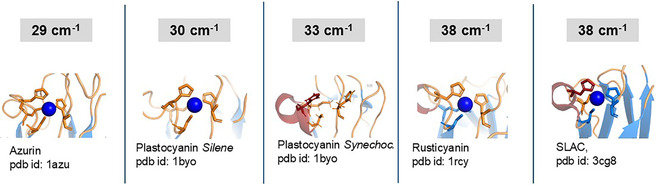
Comparison of the ligand arrangement coordinating blue copper cofactor among azurin, plastocyanin, rusticyanin and SLAC. Coordinating residues are colored according to the secondary structure elements: orange – loop, red – α−helix and blue –β‐sheet. The wavenumbers correspond to the low‐frequency modes. The values for azurin and SLAC refer to this work, plastocyanin from *Silene* [18], plastocyanin from *Synechococcus* sp (note that Cu ion is missing in the crystal structure) [16] and rusticyanin [17].

Having identified the dominant modes in low‐frequency modes in SLAC (38 cm^−1^) and azurin (29 cm^−1^), we next considered their possible assignment. Such modes are not accessible by conventional RR spectroscopy, and their molecular origin has not yet been firmly established. To date, similar low‐frequency oscillations have been detected only in a few ultrafast spectroscopic studies, notably a 38 cm^−1^ mode in rusticyanin [17], and a 30 cm^−1^ mode in plastocyanin from *Silene pratensis* [18], and 33 cm^−1^ mode in plastocyanin from *Synechococcus* sp [16]. mode in plastocyanin. These features have been hypothesized to reflect collective protein motions reminiscent of a boson peak indicating a fingerprint of an amorphous system possibly involving the whole protein skeleton [17]. The observation of a pronounced frequency shift of approximately 9 cm^−1^ between SLAC and azurin, together with a clear difference in their relative amplitudes, provides an opportunity to assess whether structural organization beyond the first coordination sphere modulates the energetic landscape governing these collective motions. In SLAC, two coordinating ligands: His231 and Cys288 are embedded within β‐sheet and His293 is embedded within α‐helix, whereas in azurin, all coordinating ligands originate from loop regions (Figure 8). A similar structural motif to SLAC is observed in rusticyanin, which exhibits a dominant low frequency mode identical to that of SLAC and features a coordinating Cys138 and Met148 located within β‐sheet. In contrast, plastocyanin, which displays a low‐frequency mode at 30 cm^−1^ shares a comparable loop‐based coordination environment (Figure 8). Interestingly, plastocyanin from *Synechococcus* sp. exhibiting slightly higher frequency (33 cm^−1^) contains conserved coordinating His86 within an α‐helix. Taken together, these correlations suggest that differences in protein rigidity and secondary‐structure embedding beyond the ligand coordination contribute to the modulation of ultrafast energy relaxation pathways, even when first‐sphere metal coordination is conserved.

## 3 Conclusion

Ultrafast pump‐probe measurements have been employed to investigate differences in excited state dynamics of two evolutionarily divergent blue copper proteins, SLAC and azurin, using ~80 fs visible excitation pluses. In SLAC, the excitation into both, the intense Cys→Cu(II) LMCT transition and a weaker d–d transition reveals a reproducible low‐frequency modulation at ~38 cm^−1^. The identical frequency, phase, and damping behavior for these two distinct excitation pathways indicates that the observed dynamics are not associated with band‐specific vibronic structure or optical artifacts. Instead, it reflects nuclear motion evolving on a common potential energy surface following rapid (<100 fs) electronic relaxation. The data further suggest that photoexcitation impulsively launches ballistic motion on the excited‐state surface, which is subsequently projected onto a nearly parallel coordinate in the ground (or mixed) electronic manifold, allowing the coherence to survive electronic relaxation. In contrast, azurin exhibits a lower‐frequency (~29 cm^−1^) mode that is critically damped, consistent with stronger coupling of the same collective coordinate to the protein bath. Correlating these dynamical differences with the structural arrangement of coordinating ligands to copper cofactor provides additional insights. In SLAC, coordinating residues are embedded in β‐sheet and α−helical elements, imparting increased mechanical rigidity to the Cu site and supporting underdamped, oscillatory motion. By contrast, in azurin, the coordinating ligands are part of flexible loops, resulting in reduced structural constraint and more strongly damped dynamics. This correlation is further supported by structural and spectroscopic data reported for plastocyanin and rusticyanin.

Together, these results reveal that low‐frequency vibrational dynamics in blue copper proteins are governed not only by the immediate metal coordination sphere but also by how the copper is embedded within the surrounding protein scaffold. These observations support a model in which photoexcitation impulsively launches motion along a collective Cu–protein vibrational coordinate whose frequency and damping are encoded by the immediate protein architecture. In SLAC, this coordinate supports ballistic, weakly damped motion that survives electronic relaxation, whereas in azurin it is strongly damped by structural flexibility. These observations highlight how protein mechanics can structurally control ultrafast nuclear dynamics at metalloprotein active sites.

## 4 Materials and Methods

### 4.1 Preparation of SLAC and Azurin Samples

Wild type SLAC has been expressed and purified as previously described [12]. Azurin, purchased from Sigma Aldrich [40] was delivered as lyophilized powder, which was reconstituted into protein solution buffered with ammonium acetate by adding H_2_O to a final concentration of 770 µM.

### 4.2 UV/Vis and RR Spectroscopy

UV/Vis spectra of ca. 500 µM (SLAC) and 770 µM (Azurin) in phosphate buffer at pH 7,5 (SLAC) and ammonium acetate (Azurin) and room temperature were recorded using Cary3500 Compact UV–Vis (Agilent). Resonance Raman spectra of the same protein solution were recorded using 594 nm laser excitation and a Confocal Raman Spectrometer (Spectroscopy&Imaging GmbH Germany) equipped with a 750 mm focal length monochromator and a thermoelectrically‐cooled back‐illuminated deep depletion CCD detector, using 1500  grooves/mm gratings. The samples were illuminated with a laser power of 5 mW and scattering was collected in back‐scattering geometry. RR spectra were accumulated 10 times for 90 s, and averaged. Baseline was subtracted by fitting a polynomial fit line.

### 4.3 TAS

Ultrafast visible‐pump/NIR‐probe femtosecond spectroscopy was performed by means of a femtosecond laser system described elsewhere [41, 42]. Briefly, two beams with average powers of 1.1 W and 330 mW were derived from a Ti:sapphire oscillator–regenerative amplifier front‐end laser system (Newport Spectra‐Physics Solstice Ace), delivering 800 nm–centered, 60 fs pulses at a repetition rate of 1 kHz. Each beam was used to synchronously pumped a commercial optical parametric amplifier (TOPAS prime, Light Conversion). A small fraction of the signal output provided the low‐power pumped TOPAS, tuned to 1250 nm, was focused into a rotating CaF_2_ substrate to generate a white‐light continuum (WLC) probe with a usable spectral bandwidth spanning 380–950 nm. Pump pulses were generated via nonlinear frequency conversion of the signal output from the high‐power TOPAS: 620 and 750 nm pulses were obtained by second‐harmonic generation of the signal, while 583 nm pulses were produced by sum‐frequency mixing of the 800 nm fundamental with idler. The probe beam was sent through a motorized delay stage to control the timing of the probe pulses relative to the pump pulses. The WLC beam was then focused into the sample and later steered to a polychromator equipped with a CCD array detector. The pump beam was focused into the sample using a fuzed‐silica lens with a focal length of 400 mm at an angle of 5° relative to the probe beam. To ensure homogeneous excitation, the pump focus was positioned slightly behind the sample, resulting in a spot diameter (~400 μm) larger than that of the probe. The sample solution was held in a commercial sample cell (Hellma, QS) with an optical pathlength of 1 mm. Pulse durations were characterized by intensity autocorrelation measurements. Assuming Gaussian pulse shapes, pulse durations of 71, 74, and 81 fs were obtained for beams with central wavelengths of 583, 620, and 750 nm respectively.

## Supporting Information

Additional supporting information can be found online in the Supporting Information section.

**Additional supporting information can be found online in the Supporting Information section. Supporting Fig. 1**: Structural alignment of azurin's (orange, pdb id 1azu) and SLAC's (blue, pdb id 3cg8) cupredoxin‐fold domains prepared in pymol using the command “cealign”. The structural similarity between the two domains has been determined to an RMSD of 5.5. **Supporting Fig. 2**: Contour plot representation of the transient absorption spectra of azurin illustrating the group velocity dispersion (GVD) correction. **(a)** Raw experimental data exhibiting a *chirped* time‐zero. The dashed black line tracks the arrival time of the probe pulse as a function of wavelength. **(b)** Dispersion‐corrected data where the coherent spike and initial excitation are aligned to *t* = 0 across the entire spectral range (400–950 nm). **Supporting Fig. 3**: Results of the Hankel Singular Value Decomposition (HSVD) performed on azurin's transient absorption spectra dataset. **(a)** Total HSVD fit to the experimental data. **(b)** Residuals remaining after the HSVD fit, showing minimal structured noise. **(c)** Contribution of the near‐zero frequency components, representing the non‐oscillatory population kinetics (incoherent decay). **(d)** High‐frequency components extracted from the fit, highlighting coherent vibrational oscillations and wavepacket dynamics. Vertical dash‐dotted lines indicate π phase flips occurring at the peaks of the ground‐state absorption bands. **Supporting Fig. 4**: Selected transient kinetic traces at selected probe wavelengths (454–720 nm) of azurin after optical excitation illustrating the HSVD decomposition process. (a) Raw transient absorption data (open circles) overlaid with the reconstructed near‐zero frequency components (solid black lines). These components represent the incoherent population dynamics. (b) High‐frequency oscillatory components (solid black lines) overlaid on the data residuals (open circles) obtained after subtracting the near‐zero frequency components. The high‐frequency fit accurately captures the vibrational wavepacket dynamics across the entire spectral range. Supporting Fig. 5: a) Transient absorption kinetics of SLAC at the indicated wavelengths, with a b) zoom in of a highlighted area. Points show the experimental data and black lines represent the fit. Positive and negative contributions are marked in light red and light blue, respectively. In b) red points refer to the experimental data after 583 nm excitation, blue points refer to the experimental data after 750 nm excitation. The kinetic trace recorded at 870 nm have been multiplied by a factor 3 to enhance visibility. **Supporting Fig. 6**: Validation of extracted vibrational frequencies. The blue solid line represents the Fast Fourier Transform of the high‐frequency residuals, providing a broad overview of the spectral density. The orange histogram represents the distribution of frequencies retrieved directly from the HSVD analysis across all probe wavelengths. The clear overlap between the FFT peaks and the highest counts in the HSVD histogram (notably near 35 cm^−1^, 85 cm^−1^, and 115 cm^−1^) confirms the statistical significance of these modes. Supporting Fig. 7: Spectrally resolved amplitude and phase retrieved from Fourier transformation of the residuals after removing slow varying background, in SLAC. Blue traces (left axis) show the signal amplitude, while red traces (right axis) show the corresponding phase (in radians). Lighter‐colored traces represent data obtained after optical excitation at 583 nm, while darker traces represent data obtained after optical excitation at 750 nm. Shaded gray regions indicate spectral intervals excluded from the analysis due to intense pump scatter. *Top panel*: Amplitude and phase corresponding to the optically induced DOD modulation at 38 cm^−1^. *Bottom panel*: Amplitude and phase corresponding to the optically induced DOD modulation at 190 cm^−1^. Supporting Fig. 8: Spectrally resolved amplitude and phase retrieved from Fourier transformation of the residuals after removing slow varying background, in azurin. Blue traces (left axis) show the signal amplitude, while red traces (right axis) show the corresponding phase (in radians). *Top panel*: Amplitude and phase corresponding to the optically induced DOD modulation at 29 cm^−1^. *Middle panel*: Amplitude and phase corresponding to the optically induced DOD modulation at 82 cm^−1^. *Bottom panel*: Amplitude and phase corresponding to the optically induced DOD modulation at 110 cm^−1^. **Supporting Fig. 9**: (a) vis‐nIR spectra of WT‐SLAC, used for fitting the GSB. (b) Target fit vis‐nIR spectra of induced absorption states of WT SLAC after 580 nm pump. (c) Target fit vis‐nIR spectra of induced absorption states of SLAC after 750 nm pump. (d) Corresponding population time‐traces retrieved from target fit. In blue: MC d states, green: LF states and dark yellow: vibrationally excited GS. (e) Circles: SLAC experimental ΔOD time traces after 583 nm pump at selected probe wavelengths. Solid lines: corresponding target fits. **Supporting Fig. 10**: (a) Population time‐traces retrieved from target fit analysis of azurin's data. b) vis‐nIR spectra of azurin with the target fit vis‐nIR spectra of induced absorption states of azurin. In blue: MC d states, green: LF states and dark yellow: vibrationally excited GS, (c) Circles: azurin experimental DOD time traces at selected probe wavelengths. Solid lines: corresponding target fits. **Supporting Fig. 11**: False color contour plots of the residuals obtained from the global target analysis of azurin transient absorption data. (a) Residuals resulting from the 4‐state kinetic model proposed by Nagasawa et al. (b) Residuals resulting from a simplified 3‐state model. The color scale represents the difference between the experimental data and the fit, with the white vertical bar indicating excluded data due to pump scatter.

## Funding

This study was supported by Deutsche Forschungsgemeinschaft (KI2279/1‐1, 509895886).

## Conflicts of Interest

The authors declare no conflicts of interest.

## Supporting information

Supplementary Material

## Data Availability

The data that support the findings of this study are available from the corresponding author upon reasonable request.
