# Supplementary material for: Transient Absorption Spectroscopy of Blue Copper Sites in Divergent Protein Folds: Azurin Versus Multicopper Oxidase
Source: Chemphyschem. 2026 Apr 28;27(8):e202500924. doi: 10.1002/cphc.202500924 (PMC13123458; doi:10.1002/cphc.202500924)
Supplement: Supplementary file 1 — Supplementary Material [file CPHC-27-e202500924-s001.pdf]

Supporting Information to:

Transient Absorption Spectroscopy of Blue Copper Sites in Divergent Protein Folds: Azurin vs. Multicopper Oxidase

Luis Ignacio Domenianni,*[a] and Patrycja Kielb*[a,b]

[a] Dr. Luis I. Domenianni, Jun.-Prof. Dr. Patrycja Kielb
Clausius Institute of Physical and Theoretical Chemistry
University of Bonn
Wegelerstrasse 12, 53115 Bonn, Germany
E-mail: ldomenia@uni-bonn.de, kielb@uni-bonn.de

[b] Jun.-Prof. Dr. Patrycja Kielb
Transdisciplinary Research Area 'Building Blocks of Matter and Fundamental Interactions'
University of Bonn
53115 Bonn, Germany
Email: kielb@uni-bonn.de

Content:

Fig. S1. Structural alignment of azurin and SLAC.

Fig. S2. Group velocity dispersion correction of TAS data exemplified on the TAS spectra of azurin.

Fig. S3. Results of the Hankel Singular Value Decomposition (HSVD) performed on azurin's transient absorption spectra dataset.

Fig. S4. Transient kinetic traces of azurin at selected probe wavelengths.

Fig. S5. Transient absorption kinetics of SLAC at selected probe wavelengths.

Fig. S6. Validation of extracted vibrational frequencies.

Fig. S7. Amplitude and phase analysis of Fourier transformed transient absorption spectra of SLAC.

Fig. S8. Amplitude and phase analysis of Fourier transformed transient absorption spectra of azurin.

Fig. S9. Target fit analysis of transient absorption spectra of SLAC.

Fig. S10. Target fit analysis of transient absorption spectra of azurin.

Fig. S11. False color contour plots of the residuals obtained from the global target analysis of azurin transient absorption data, 4-state vs. 3-state kinetic model.

1. Structural comparison of SLAC and azurin

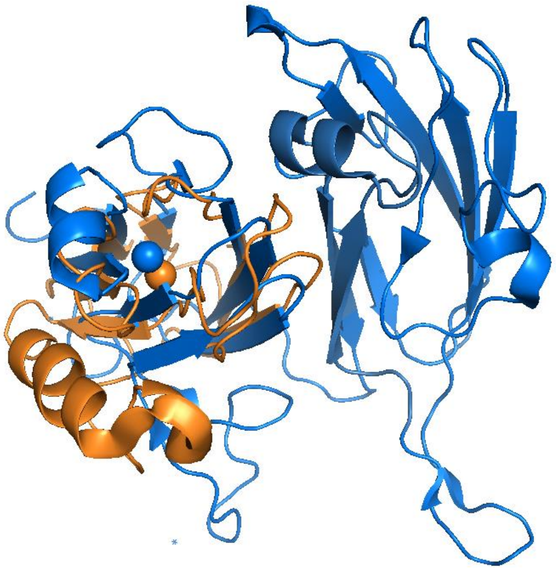

Figure. S1. Structural alignment of azurin's (orange, pdb id 1azu) and SLAC's (blue, pdb id 3cg8) cupredoxin-fold domains prepared in pymol using the command 'cealign'. The structural similarity between the two domains has been determined to an RMSD of 5.5.

2. TAS Data Treatment

2.1. Dispersion correction

In ultrafast pump-probe experiments the supercontinuum white-light probe undergoes significant Group Velocity Dispersion (GVD) as it passes through the generation medium (CaF_2) and the sample cuvette, causing redder photons to arrive earlier than bluer photons. The averaged raw pump–probe data was corrected for wavelength-dependent dispersion of the white-light probe. To determine the temporal offset at each probe wavelength, the transient signal of the pure solvent was measured and the temporal position of the coherent solvent response was used to extract the delay shift associated with each wavelength channel. The resulting wavelength-dependent delay offsets were subtracted from the experimental delay axis to compensate for probe chirp. Following this correction, the transient recorded at each wavelength (pixel) was interpolated onto a common delay grid, yielding a dispersion-corrected dataset with a single shared delay axis for all wavelengths. This correction is a prerequisite for the accurate HSVD decomposition and global target analysis performed in this study.

Figure S2 exemplifies the dispersion correction for the transient absorption spectra of azurin. SLAC was treated in analogous fashion.

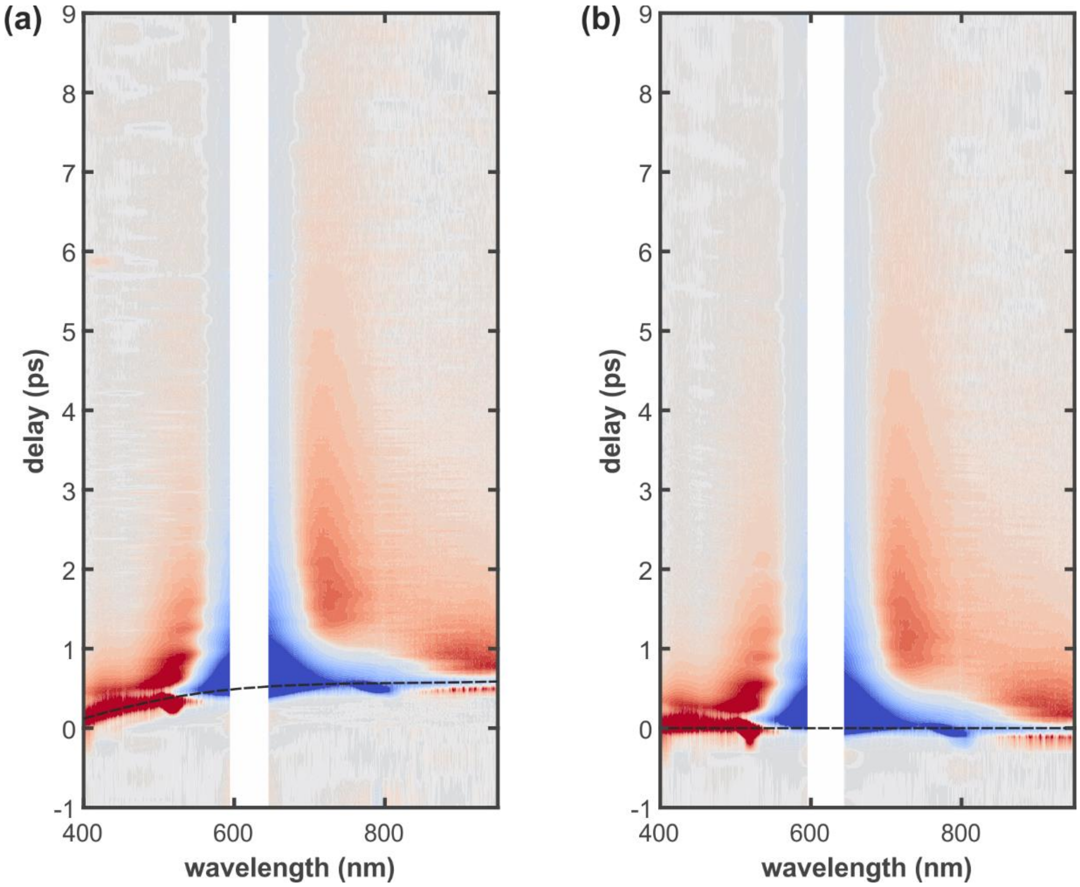

Figure S2. Contour plot representation of the transient absorption spectra of azurin illustrating the group velocity dispersion (GVD) correction. **(a)** Raw experimental data exhibiting a *chirped* time-zero. The dashed black line tracks the arrival time of the probe pulse as a function of wavelength. **(b)** Dispersion-corrected data where the coherent spike and initial excitation are aligned to t = 0 across the entire spectral range (400–950 nm).

2.2. Hankel Singular Value Decomposition fits

The transient signals, starting at 0.3 ps (to avoid any significant artifacts), were analyzed using the Hankel Singular Value Decomposition (HSVD) method. This method allows for the separation of the experimental data into distinct physical components without a predetermined kinetic model. In this approach, the time-domain signal is expressed as a sum of exponentially damped oscillatory components. The experimental time trace is arranged into a Hankel matrix constructed from the measured data points, which is then decomposed using singular value decomposition (SVD). From the truncated signal subspace, a small matrix is constructed whose eigenvalues correspond to the signal poles

$$z_k = e^{(-b_k + i\omega_k)\Delta t},$$

from which the frequencies and decay constants are directly obtained. b_k and ω_k are the damping factors and angular frequencies of sinusoid k and Δt is the constant sampling interval.

It should be noted that this procedure does not involve any explicit kinetic or physical model for the dynamics. Instead, HSVD provides a mathematical decomposition of the transient signal into a set of exponentially damped components whose parameters are determined statistically from the data. The resulting poles represent the dominant oscillatory and decaying contributions present in the measured signal rather than a predefined mechanistic model. Detailed descriptions of the HSVD formalism can be found in Refs. [1] and [2].

This process is exemplified for azurin in Figure S3. Panel (a) shows the HSVD fit of azurin transient absorption spectra. The negligible residuals (Panel b) confirm that the HSVD basis set sufficiently describes the full complexity of the experimental data. The decomposition into near-zero frequency components (Panel c) effectively captures the multi-exponential population decay and solvation dynamics. Crucially, the high-frequency components (Panel d) reveal structured, oscillatory features particularly prominent in the 500–600 nm and 700–800 nm regions during the first 1.5 ps. These oscillations are consistent with nuclear wavepacket motion. The clean separation of these components in HSVD demonstrates that while the population kinetics dominate the signal amplitude, the system exhibits significant vibrational coherence. These high-frequency oscillatory components exhibit distinct π phase flips (denoted by vertical dash-dotted lines) at specific probe wavelengths.

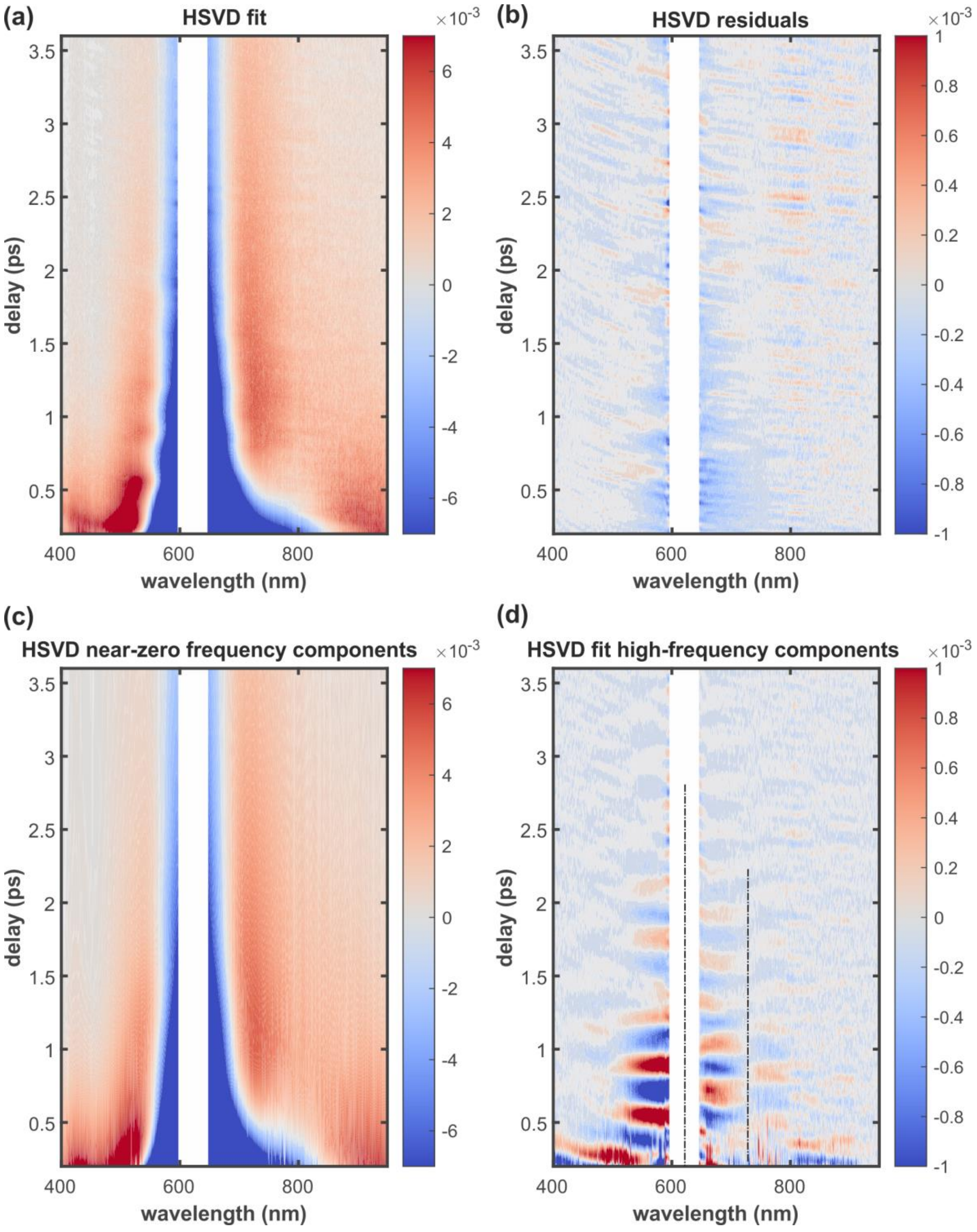

Figure S3. Results of the Hankel Singular Value Decomposition (HSVD) performed on azurin's transient absorption spectra dataset. **(a)** Total HSVD fit to the experimental data. **(b)** Residuals remaining after the HSVD fit, showing minimal structured noise. **(c)** Contribution of the near-zero frequency components, representing the non-oscillatory population kinetics (incoherent decay). **(d)** High-frequency components extracted from the fit, highlighting coherent vibrational oscillations and wavepacket dynamics. Vertical dash-dotted lines indicate π phase flips occurring at the peaks of the ground-state absorption bands.

Figure S4 provides further insight into the HSVD analysis of azurin. In Panel (a), the near-zero frequency components (representing non-oscillatory decay) provide a smooth backbone for the data. The systematic deviations of the raw data from these lines (most visible as "wiggles" in the 540–649 nm traces) correspond to the coherent vibrational information. In Panel (b), the "incoherent" background was subtracted, and the resulting residuals were overlaid with the high-frequency HSVD components. The excellent agreement between the oscillatory fit and the residuals confirms that the high-frequency components are not picking up residual population kinetics and that the HSVD accurately tracks the phase and damping of the wavepackets across the broad spectral range. This decomposition allows for the independent analysis of electronic relaxation rates (from Panel a) and nuclear vibrational frequencies (from Panel b) without the two signals interfering with one another during the optimization process.

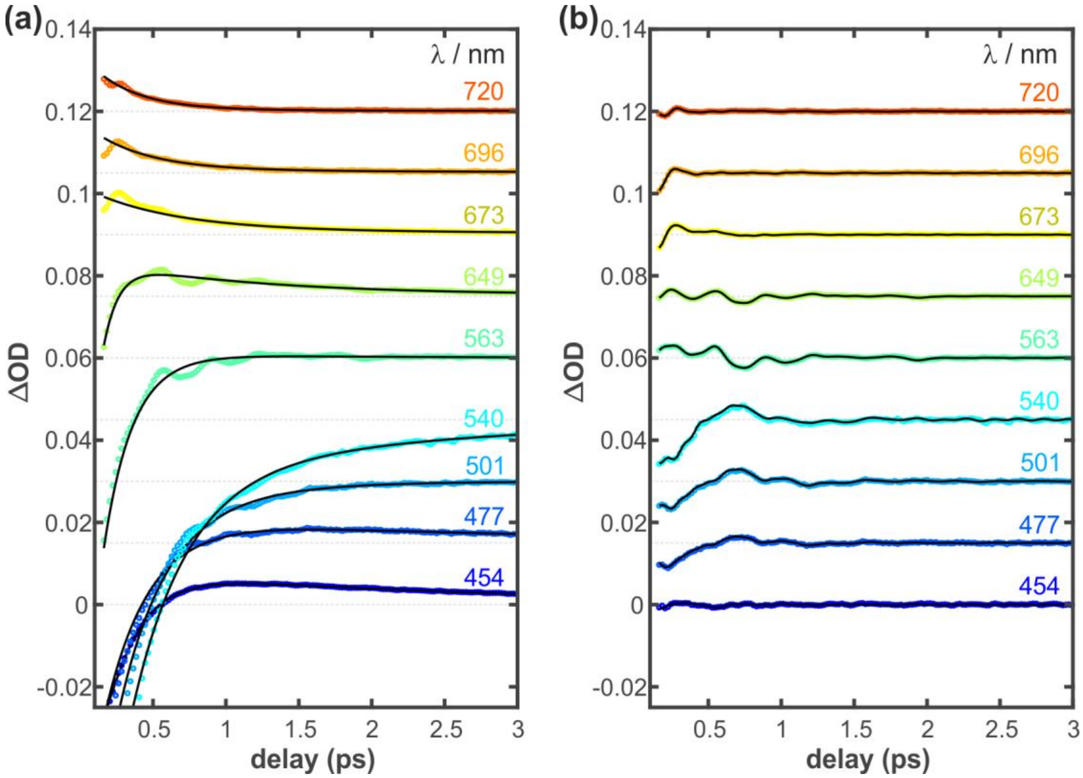

Figure S4. Selected transient kinetic traces at selected probe wavelengths (454–720 nm) of azurin after optical excitation illustrating the HSVD decomposition process. (a) Raw transient absorption data (open circles) overlaid with the reconstructed near-zero frequency components (solid black lines). These components represent the incoherent population dynamics. (b) High-frequency oscillatory components (solid black lines) overlaid on the data residuals (open circles) obtained after subtracting the near-zero frequency components. The high-frequency fit accurately captures the vibrational wavepacket dynamics across the entire spectral range.

Panel (a) of Figure S5 highlights the near-zero frequency components of the HSVD analysis of SLAC after optical excitation at 750 nm for selected probe wavelengths. Once more, the systematic deviations of the raw data from these lines correspond to the coherent vibrational information. In Panel (b), a comparison between the experimental data recorded after 583 and 750 nm excitation is highlighted**.**

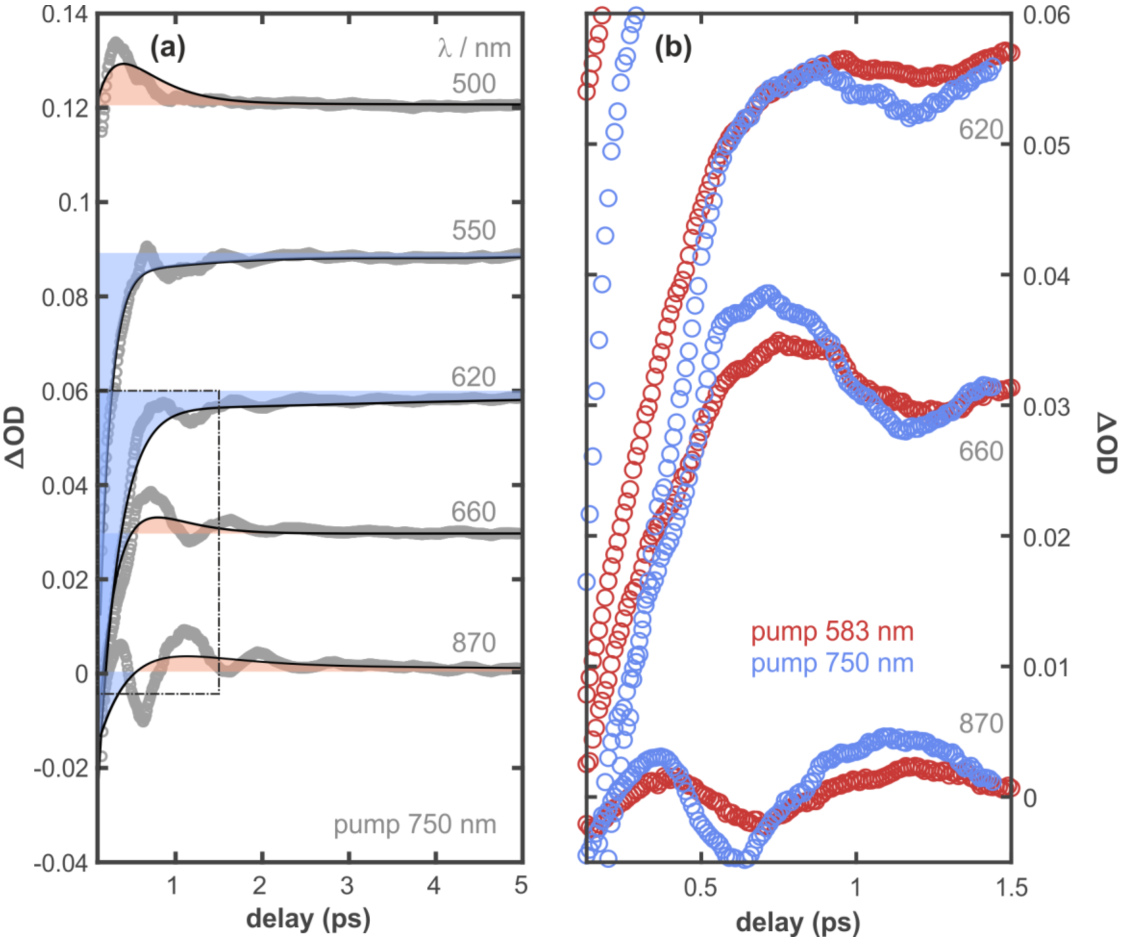

Figure S5. a) Transient absorption kinetics of SLAC at the indicated wavelengths, with a b) zoom in of a highlighted area. Points show the experimental data and black lines represent the fit. Positive and negative contributions are marked in light red and light blue, respectively. In b) red points refer to the experimental data after 583 nm excitation, blue points refer to the experimental data after 750 nm excitation. The kinetic trace recorded at 870 nm have been multiplied by a factor 3 to enhance visibility.

2.3. Frequency and phase extraction of coherent oscillations

The oscillatory component of the transient absorption signal was obtained as the difference between the measured signal and the near-zero frequency components of the HSVD fit. The remaining signal, containing the coherent modulations of the transient signal, was analyzed in the frequency domain by fast Fourier transformation (FFT). For each probe wavelength, the oscillatory time trace was multiplied by a Hann window function in order to reduce spectral leakage associated with the finite time window. The windowed signal was then zero-padded and Fourier transformed along the delay axis. Only the positive-frequency part of the spectrum was retained to obtain the single-sided amplitude spectrum. The oscillation amplitudes were obtained from the magnitude of the Fourier coefficients after normalization by the window coherent gain and the number of data points. The phase of each oscillatory component was determined from the argument of the complex Fourier coefficients and the phase profiles were unwrapped along the wavelength axis to remove discontinuities arising from the 2π periodicity of the complex phase.

To ensure the reliability of the coherent components extracted, we compared the frequency domain representation obtained via FFT with the discrete frequencies identified by the HSVD algorithm in Figure

S6. The FFT (blue curve) captures the ensemble average of the oscillations, showing several distinct peaks in the low-frequency region (0–300 cm^{-1}). These peaks likely correspond to collective modes of the azurin scaffold (see main text). The HSVD histogram (orange bars), represents the count of specific frequency components identified at individual probe wavelengths. The histogram peaks align perfectly with the FFT maxima demonstrating that the HSVD is not "over-fitting" or generating spurious frequencies, as it independently converges on the same modes identified by the FFT. The large zero frequency spike in the histogram represents the population dynamics.

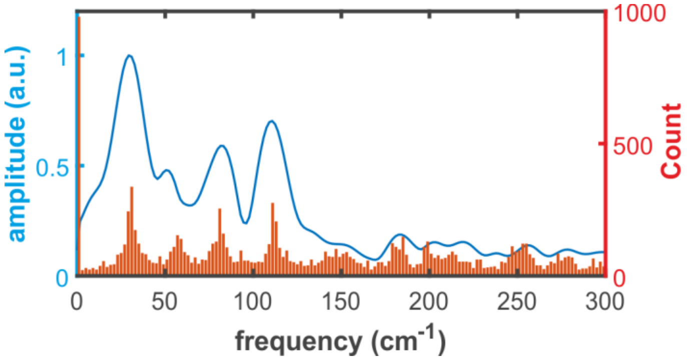

Figure S6. Validation of extracted vibrational frequencies. The blue solid line represents the Fast Fourier Transform of the high-frequency residuals, providing a broad overview of the spectral density. The orange histogram represents the distribution of frequencies retrieved directly from the HSVD analysis across all probe wavelengths. The clear overlap between the FFT peaks and the highest counts in the HSVD histogram (notably near 35 cm^{-1}, 85 cm^{-1}, and 115 cm^{-1}) confirms the statistical significance of these modes.

Figures S7 and S8 present the spectrally resolved amplitude and phase for the two dominant low-frequency modes identified in SLAC's transient spectra at 38 cm-1 and 190 cm-1 and the three dominant low-frequency modes of azurin at 29 cm^{-1}, 82 cm^{-1} and 110 cm^{-1}. Notice that below 500 nm the oscillatory amplitude extracted from the FFT analysis is negligible and no clear modulations are apparent in the time-domain data. Under these conditions, the phase becomes ill-defined and can exhibit apparent discontinuities arising from the numerical procedure rather than from a physically meaningful signal.

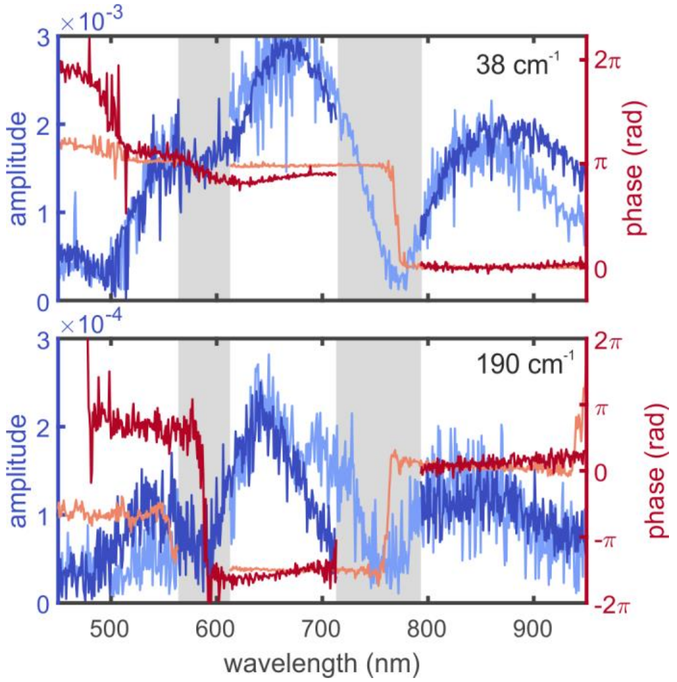

Figure S7. Spectrally resolved amplitude and phase retrieved from Fourier transformation of the residuals after removing slow varying background, in SLAC. Blue traces (left axis) show the signal amplitude, while red traces (right axis) show the corresponding phase (in radians). Lighter-colored traces represent data obtained after optical excitation at 583 nm, while darker traces represent data obtained after optical excitation at 750 nm. Shaded gray regions indicate spectral intervals excluded from the analysis due to intense pump scatter. ***Top panel*:** Amplitude and phase corresponding to the optically induced DOD modulation at 38 cm-1. ***Bottom panel*:** Amplitude and phase corresponding to the optically induced DOD modulation at 190 cm-1

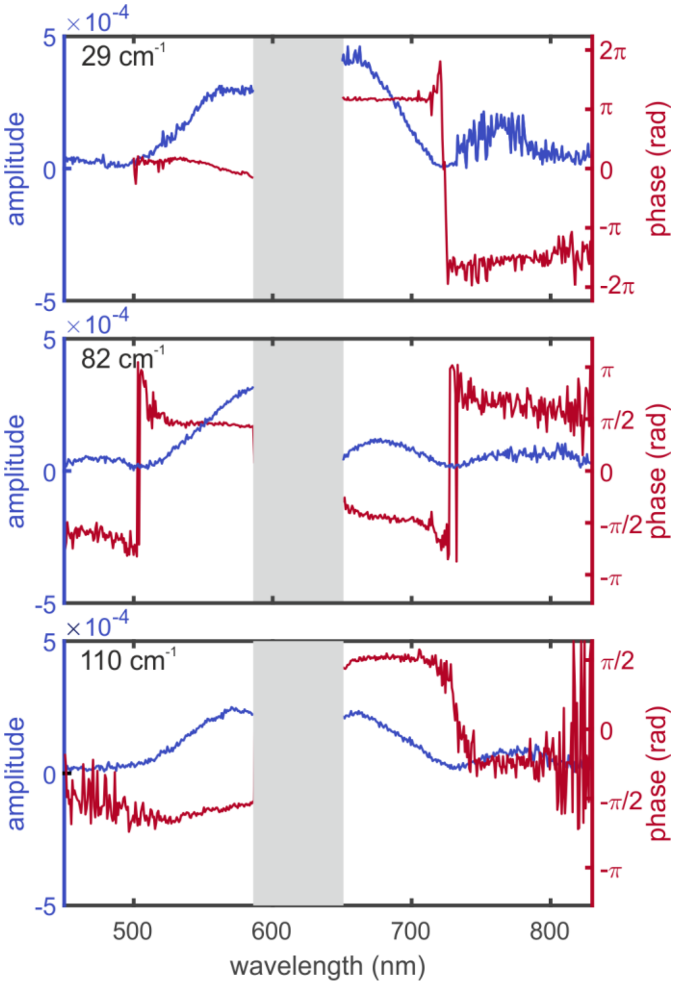

Figure S8. Spectrally resolved amplitude and phase retrieved from Fourier transformation of the residuals after removing slow varying background, in azurin. Blue traces (left axis) show the signal amplitude, while red traces (right axis) show the corresponding phase (in radians). ***Top panel*****:** Amplitude and phase corresponding to the optically induced DOD modulation at 29 cm-1. ***Middle panel*****:** Amplitude and phase corresponding to the optically induced DOD modulation at 82 cm-1. ***Bottom panel*****:** Amplitude and phase corresponding to the optically induced DOD modulation at 110 cm-1.

2.4. Kinetic modelling

Following Nagasawa et al. the pump prepares an LMCT state, that undergoes an LMCT → MC transition within our instrumental response function (~70–80 fs). Rendering the following effective mechanism

$$\mathrm{GS} \overset{h\nu}{\rightarrow} \mathrm{MC} \overset{k_1}{\rightarrow} \mathrm{LF} \overset{k_2}{\rightarrow} \mathrm{GS}^* \overset{k_3}{\rightarrow} \mathrm{GS}$$

The coupled kinetic differential equations then read

$$\frac{\mathrm{d[GS]}}{\mathrm{d}t} = k_3[\mathrm{GS}^*]$$

$$\frac{\mathrm{d[MC]}}{\mathrm{d}t} = -k_1[\mathrm{MC}]$$

$$\frac{\mathrm{d[LF]}}{\mathrm{d}t} = k_1[\mathrm{MC}] - k_2[\mathrm{LF}]$$

$$\frac{\mathrm{d[GS^*]}}{\mathrm{d}t} = k_2[\mathrm{LF}] - k_3[\mathrm{GS}^*]$$

where the brackets denote the time-dependent populations of the four states involved in the model. Analytical solutions of the rate equations yield the following population expressions

$$[\mathrm{GS}] = [\mathrm{GS}]_0 + [\mathrm{MC}]_0\left[\frac{k_2k_3e^{-k_1t}}{(k_1-k_2)(k_1-k_3)} - \frac{k_1k_3e^{-k_2t}}{(k_1-k_2)(k_2-k_3)} + \frac{k_1k_2e^{-k_3t}}{(k_1-k_3)(k_2-k_3)} - 1\right] - [\mathrm{LF}]_0\left[\frac{k_3e^{-k_2t} - k_2e^{-k_3t}}{k_2-k_3} + 1\right] - [\mathrm{GS}^*]_0(e^{-k_3t} - 1)$$

$$[\mathrm{MC}] = [\mathrm{MC}]_0\, e^{-k_1t}$$

$$[\mathrm{LF}] = [\mathrm{LF}]_0e^{-k_2t} - [\mathrm{MC}]_0\frac{k_1}{(k_1-k_2)}[e^{-k_1t} - e^{-k_2t}]$$

$$[\mathrm{GS}^*] = [\mathrm{GS}^*]_0e^{-k_3t} + [\mathrm{MC}]_0k_1k_2\left[\frac{e^{-k_1t}}{(k_1-k_2)(k_1-k_3)} - \frac{e^{-k_2t}}{(k_1-k_2)(k_2-k_3)} + \frac{e^{-k_3t}}{(k_1-k_3)(k_2-k_3)}\right] - [\mathrm{LF}]_0\frac{k_2}{(k_2-k_3)}[e^{-k_2t} - e^{-k_3t}]$$

where $[\mathrm{MC}]_0$ is the population created upon optical excitation and initial non-adiabatic relaxation from the optically prepared excited state within the experimental time-resolution. Since initially $[\mathrm{GS}^*]_0$ and $[\mathrm{LF}]_0$ are zero, the rate equations simplify to

$$[\mathrm{GS}] = [\mathrm{GS}]_0 + [\mathrm{MC}]_0\left[\frac{k_2k_3e^{-k_1t}}{(k_1-k_2)(k_1-k_3)} - \frac{k_1k_3e^{-k_2t}}{(k_1-k_2)(k_2-k_3)} + \frac{k_1k_2e^{-k_3t}}{(k_1-k_3)(k_2-k_3)} - 1\right]$$

$$[\mathrm{MC}] = [\mathrm{MC}]_0\, e^{-k_1t}$$

$$[\mathrm{LF}] = [\mathrm{MC}]_0\frac{k_1}{(k_1-k_2)}[e^{-k_2t} - e^{-k_1t}]$$

$$[\mathrm{GS}^*] = [\mathrm{MC}]_0k_1k_2\left[\frac{e^{-k_1t}}{(k_1-k_2)(k_1-k_3)} - \frac{e^{-k_2t}}{(k_1-k_2)(k_2-k_3)} + \frac{e^{-k_3t}}{(k_1-k_3)(k_2-k_3)}\right]$$

2.5. Target analysis

Prior to kinetic modelling, the high-frequency oscillatory components identified via HSVD were removed in order to isolate the population dynamics.

The experimental transient absorption data were organized in a matrix

$$\mathbf{\Delta OD}_{m\times p} = \begin{pmatrix} \Delta\mathrm{OD}(\lambda_1, t_1) & \Delta\mathrm{OD}(\lambda_1, t_2) & \cdots & \Delta\mathrm{OD}(\lambda_1, t_p) \\ \Delta\mathrm{OD}(\lambda_2, t_1) & \Delta\mathrm{OD}(\lambda_2, t_2) & \cdots & \Delta\mathrm{OD}(\lambda_2, t_p) \\ \vdots & \vdots & \ddots & \vdots \\ \Delta\mathrm{OD}(\lambda_m, t_1) & \Delta\mathrm{OD}(\lambda_m, t_2) & \cdots & \Delta\mathrm{OD}(\lambda_m, t_p) \end{pmatrix}$$

where *m* and *p* denote the number of measured probe wavelengths λ and time delays *t*, respectively, and the matrix elements correspond to the pump-induced change in optical density any given λ_i and t_j.

In the absence of significant dynamical spectral shifts, the measured signal can be described as a bilinear superposition of spectral and kinetic contributions according to Lambert–Beer's law,

$$\Delta\mathrm{OD}(\lambda, t) = \sum_{i=1}^{n} \epsilon_i(\lambda, t)\Delta c_i(t) \approx \sum_{i=1}^{n} \epsilon_i(\lambda)\Delta c_i(t)$$

where $\epsilon_i(\lambda)$ represents the absorption spectral profile associated with species *i*, and $\Delta c_i(t)$ is the corresponding optically induced population change as a function of time.

In matrix form,

$$\mathbf{\Delta OD}_{m\times p} = \boldsymbol{\epsilon}_{m\times n} \cdot \mathbf{\Delta c}_{n\times p} = \begin{pmatrix} \epsilon_1(\lambda_1) & \epsilon_2(\lambda_1) & \cdots & \epsilon_n(\lambda_1) \\ \epsilon_1(\lambda_2) & \epsilon_2(\lambda_2) & \cdots & \epsilon_n(\lambda_2) \\ \vdots & \vdots & \ddots & \vdots \\ \epsilon_1(\lambda_m) & \epsilon_2(\lambda_m) & \cdots & \epsilon_n(\lambda_m) \end{pmatrix} \cdot \begin{pmatrix} \Delta\mathrm{c}_1(t_1) & \Delta\mathrm{c}_1(t_2) & \cdots & \Delta\mathrm{c}_1(t_p) \\ \Delta\mathrm{c}_2(t_1) & \Delta\mathrm{c}_2(t_2) & \cdots & \Delta\mathrm{c}_2(t_p) \\ \vdots & \vdots & \ddots & \vdots \\ \Delta\mathrm{c}_n(t_1) & \Delta\mathrm{c}_n(t_2) & \cdots & \Delta\mathrm{c}_n(t_p) \end{pmatrix}$$

The ground-state bleach (GSB) contribution can be determined independently from the stationary absorption spectrum of the ground state. Consequently, the spectral matrix can be separated into known GSB and unknown induced-absorption (IA) components

$$\boldsymbol{\epsilon}_{m\times n} = \left[\boldsymbol{\epsilon}_{m\times 1}^{\mathrm{GSB}} \quad \boldsymbol{\epsilon}_{m\times(n-1)}^{\mathrm{IA}}\right],$$

thereby reducing the number of unknown spectral profiles to the excited-state and product spectra. Further numerical insights into the fitting procedure can be found in the SI of Ref. [3].

The time-dependent population matrix was calculated from the analytical solutions of the kinetic rate equations derived from the mechanistic model. The unknown IA spectra and kinetic rate constants were determined by minimizing the squared deviation between experimental and reconstructed data:

The optimization procedure was carried out iteratively by updating the kinetic parameters and recalculating the spectral profiles using a pseudo-inverse solution for the spectral matrix until convergence was reached.

Figure S9 illustrates the target analysis of the SLAC transient absorption spectra following excitation at 583 nm and 750 nm. These results were obtained after removing high-frequency components identified via HSVD filtering to isolate the relevant kinetic traces.

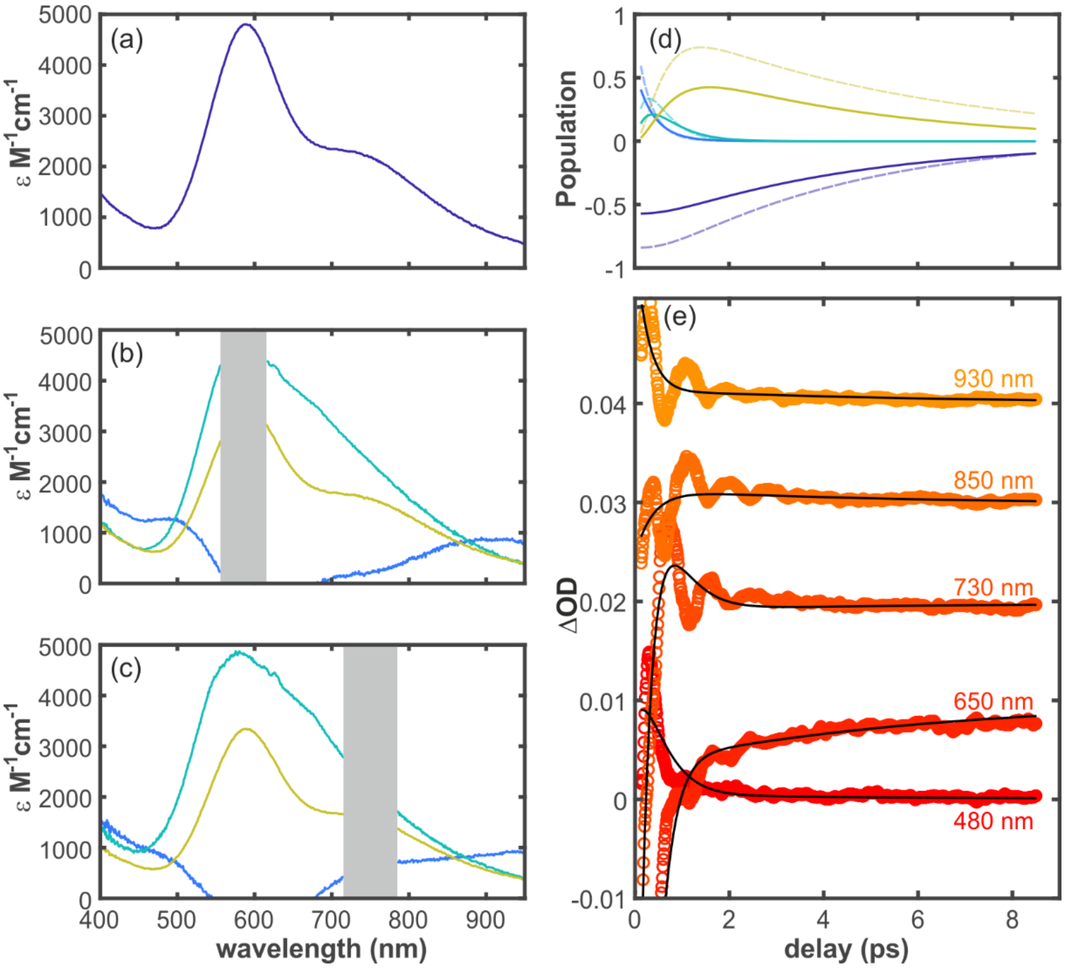

Figure S9. (a) vis-nIR spectra of WT-SLAC, used for fitting the GSB. (b) Target fit vis-nIR spectra of induced absorption states of WT SLAC after 580 nm pump. (c) Target fit vis-nIR spectra of induced absorption states of SLAC after 750 nm pump. (d) Corresponding population time-traces retrieved from target fit. In blue: MC d states, green: LF states and dark yellow: vibrationally excited GS. (e) Circles: SLAC experimental ΔOD time traces after 583 nm pump at selected probe wavelengths. Solid lines: corresponding target fits.

Figure S10 presents the results of the same aforementioned target fit model for the population dynamic components of the transient absorption spectra of azurin recorded after excitation at 620 nm.

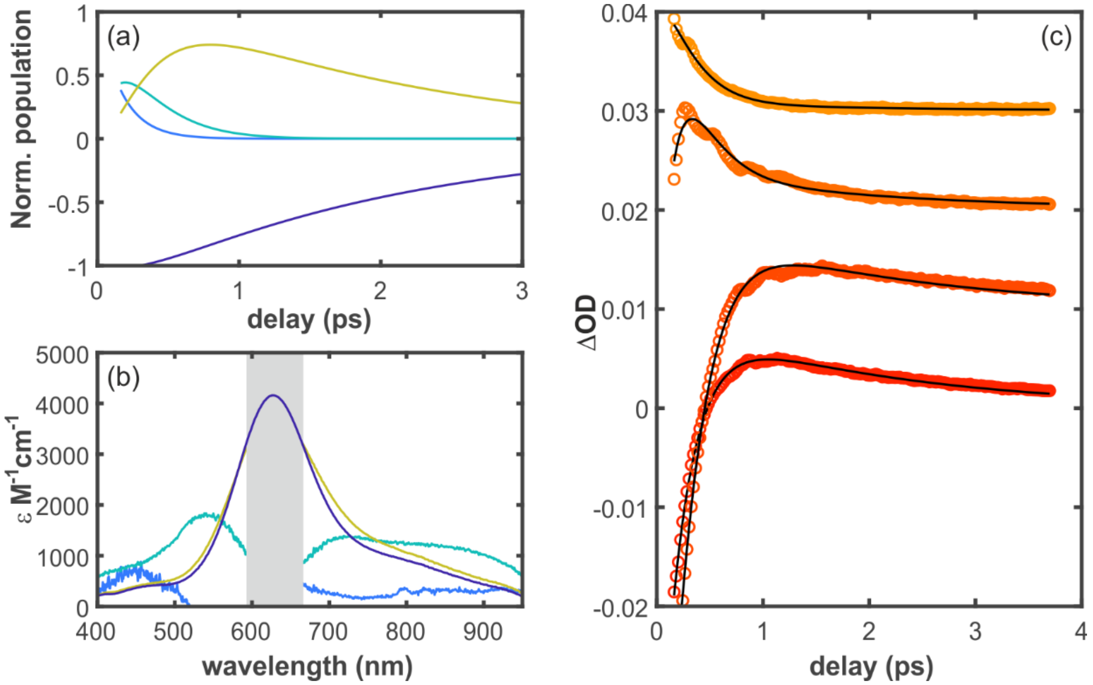

Figure S10. (a) Population time-traces retrieved from target fit analysis of azurin's data. b) vis-nIR spectra of azurin with the target fit vis-nIR spectra of induced absorption states of azurin. In blue: MC d states, green: LF states and dark yellow: vibrationally excited GS, (c) Circles: azurin experimental DOD time traces at selected probe wavelengths. Solid lines: corresponding target fits.

The necessity of a four-state model was tested to mitigate the risk of overfitting. A secondary target fit analysis was performed for both azurin and SLAC. This involved constraining the system to a three-state architecture to determine if the additional parameters significantly improved the goodness-of-fit or if a more parsimonious model remained statistically viable. A comparative analysis of the fit residuals is presented as example in Figure S11 for a target fit of azurin´s population dynamics, after removal of the high-frequency components. This analysis reveals significant differences in the performance of the two kinetic models. The 4-state Nagasawa model (a) yields a relatively homogeneous distribution of residuals across the probed spectral window (400–950 nm) and temporal range (0–3.5 ps). While some minor structured noise persists at early delay times (<0.5 ps) near 600 nm, the overall lack of coherent features suggests that the 4-state model effectively captures the primary excited-state dynamics and vibrational cooling pathways of the azurin system. In contrast, the 3-state model (b) exhibits pronounced systematic residuals, particularly in the 500–600 nm region during the first picosecond. These structured red and blue features indicate a failure of the 3-state model to account for the evolution of the transient signal, likely due to the omission of intermediate relaxation states. The superior fit of the 4-state model, as evidenced by the reduced magnitude and randomness of its residuals, justifies the inclusion of additional kinetic complexity to accurately describe the photophysical behavior of the protein.

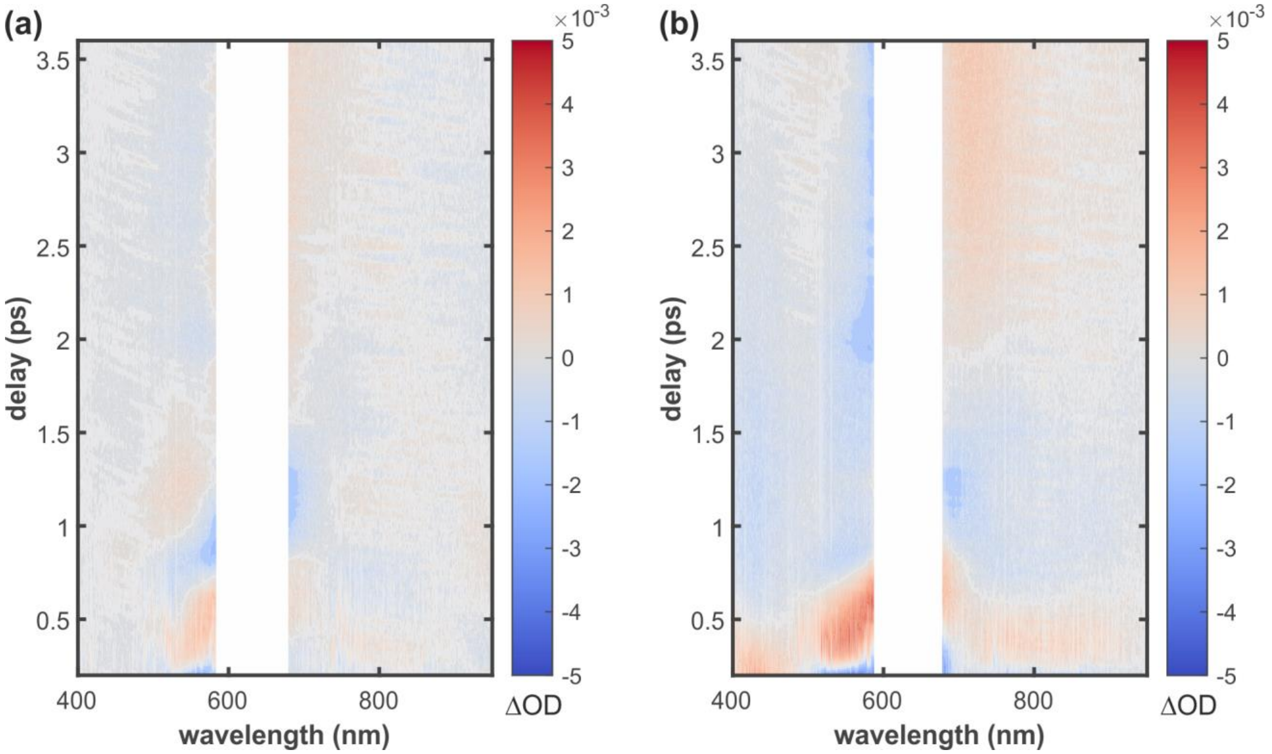

Figure S11. False color contour plots of the residuals obtained from the global target analysis of azurin transient absorption data. (a) Residuals resulting from the 4-state kinetic model proposed by Nagasawa et al. (b) Residuals resulting from a simplified 3-state model. The color scale represents the difference between the experimental data and the fit, with the white vertical bar indicating excluded data due to pump scatter.

2 References

[1] W. Pijnappel, A. Van Den Boogaart, R. De Beer, D. Van Ormondt, D. Journal of Magnetic Resonance 1992, 97, 122 – 134.

[2] H. Barkhuijsen, R. De Beer, D. Van Ormondt, Journal of Magnetic Resonance 1987, 73, 553 – 557.

[3] J. Schmidt, L. Domenianni, M. Leuschner, A. Gansäuer, P. Vöhringer, Angewandte Chemie - International Edition 2023, 62 (35). https://doi.org/10.1002/anie.202307178.
